# Supplementary material for: First Synthesis of DBU-Conjugated Cationic Carbohydrate Derivatives and Investigation of Their Antibacterial and Antifungal Activity
Source: Int J Mol Sci. 2023 Feb 10;24(4):3550. doi: 10.3390/ijms24043550 (PMC9968064; doi:10.3390/ijms24043550)
Supplement: Supplementary file 1 [file ijms-24-03550-s001.zip › ijms-2161839-supplementary.pdf]

Supporting Information

First synthesis of DBU-conjugated cationic carbohydrate derivatives and investigation of their antibacterial and antifungal activity

Fruzsina Demeter [1], Patrik Török [1], Alexandra Kiss [2], Richárd Kovásznai-Oláh [2], Zsuzsa Máthéné Szigeti [2], Viktória Baksa [2], Fruzsina Kovács [3,4], Noémi Balla [3,4], Ferenc Fenyvesi [5], Judit Váradi [5], Anikó Borbás [1,*] and Mihály Herczeg [1,*]

[1]*Department of Pharmaceutical Chemistry, Faculty of Pharmacy, University of Debrecen, Egyetem tér 1, H-4032 Debrecen, Hungary*

[2]*Institute of Biotechnology, Faculty of Science and Technology, University of Debrecen, Egyetem tér 1, H-4032, Debrecen, Hungary.*

[3]*Department of Medical Microbiology, Faculty of Medicine, University of Debrecen, 4032 Debrecen, Hungary;*

[4]*Doctoral School of Pharmaceutical Sciences, University of Debrecen, 4032 Debrecen, Hungary*

[5]*Department of Pharmaceutical Technology, Faculty of Pharmacy, University of Debrecen, Nagyerdei Körút 98., H-4032, Debrecen, Hungary;*

E-mail: borbas.aniko@pharm.unideb.hu; herczeg.mihaly@pharm.unideb.hu

Table of Contents

^{1}H and ^{13}C NMR spectra of the synthesized compounds **2**

^{1}H and ^{13}C NMR spectra of compound **39**

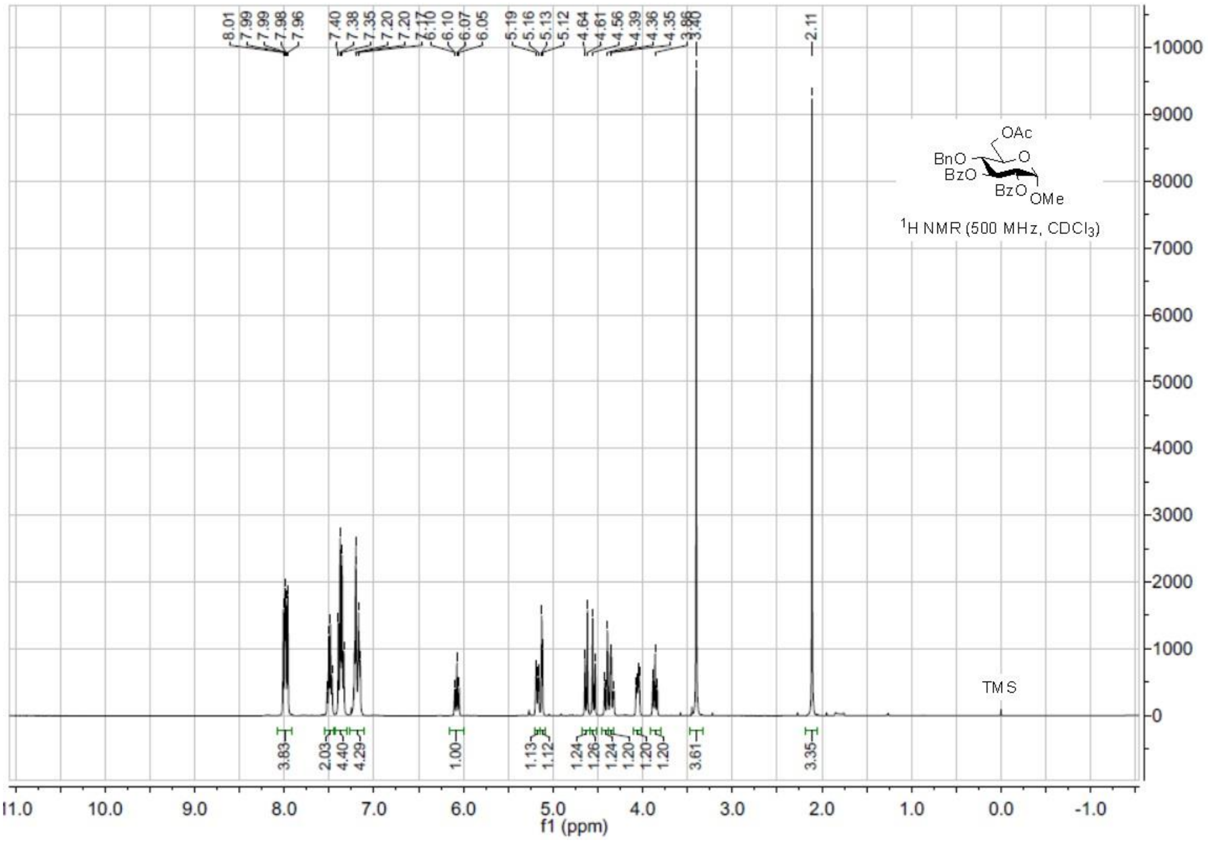

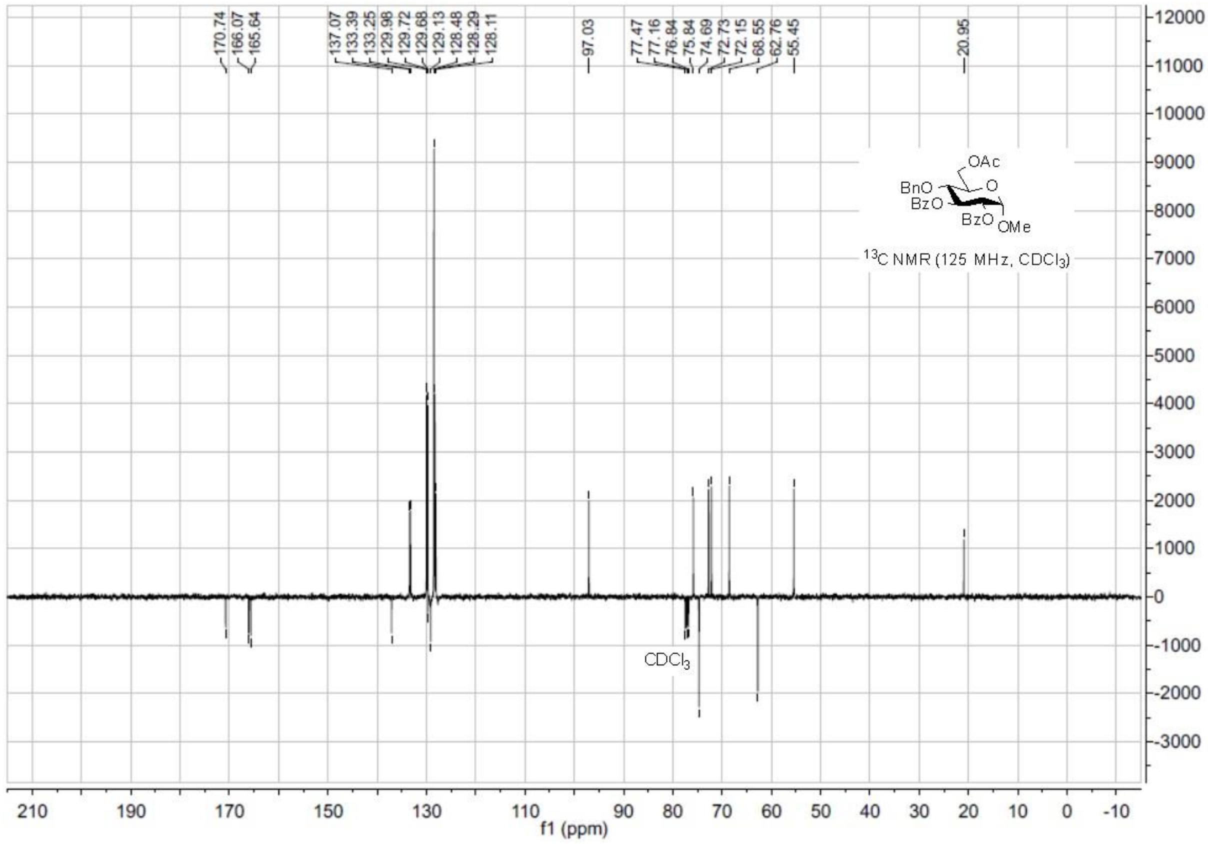

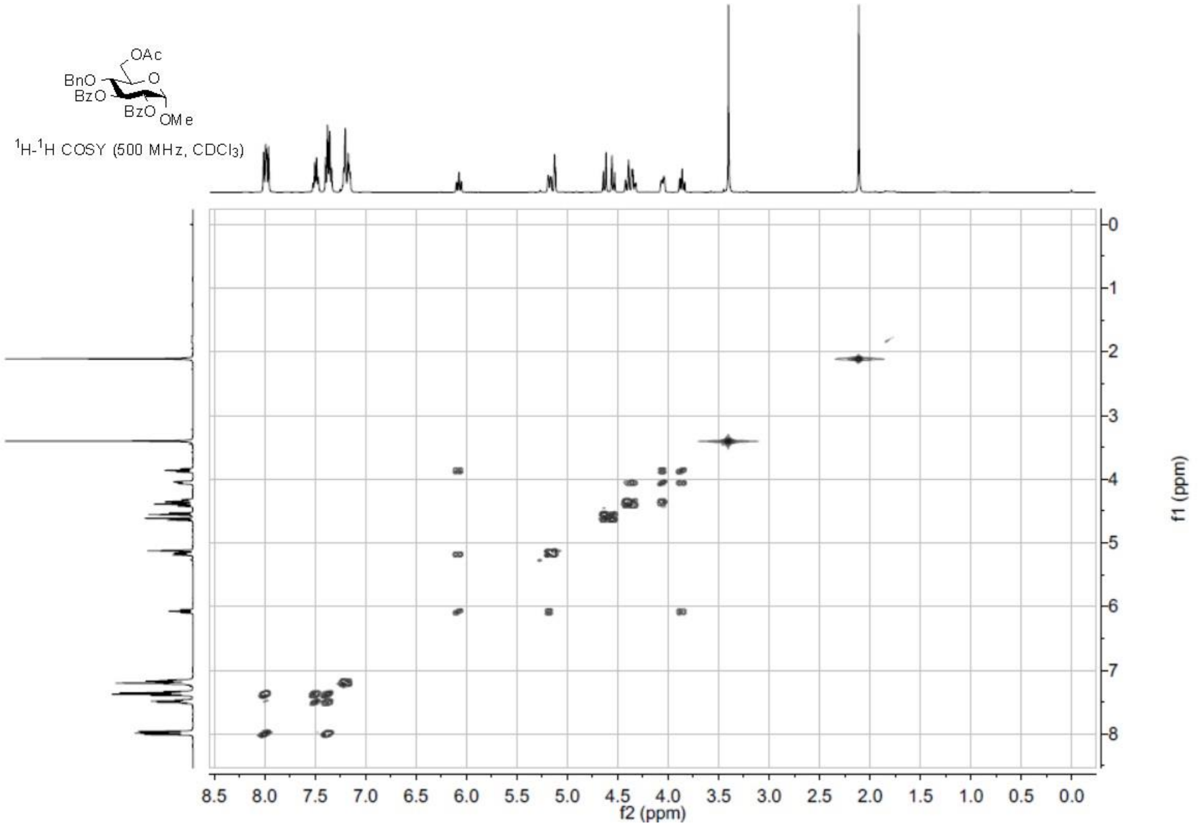
OAc
BnO
O
BzO
BzO
OMe
^{1}H-^{1}H COSY (500 MHz, CDCl$_3$)
f2 (ppm)
f1 (ppm)

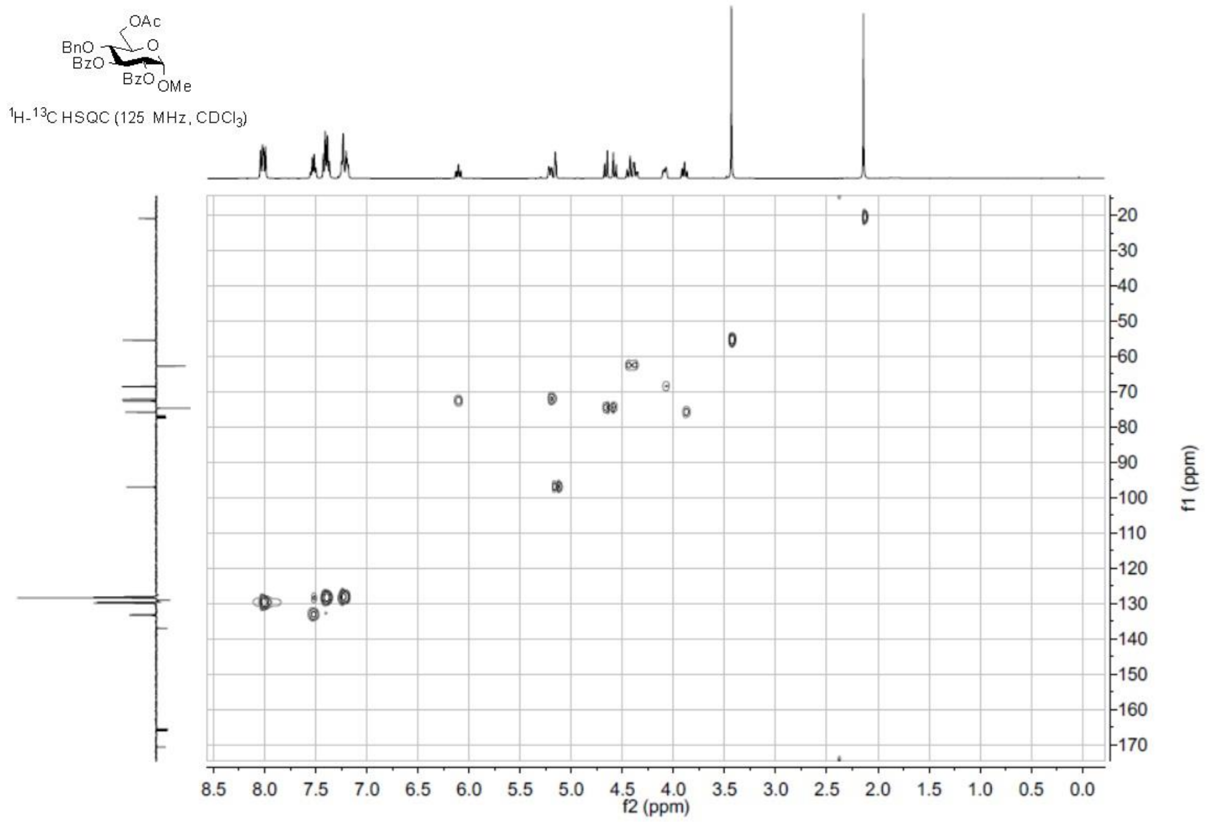
OAc
BnO
O
BzO
BzO
OMe
^{1}H-^{13}C HSQC (125 MHz, CDCl$_3$)
f2 (ppm)
f1 (ppm)

^{1}H and ^{13}C NMR spectra of compound **43**

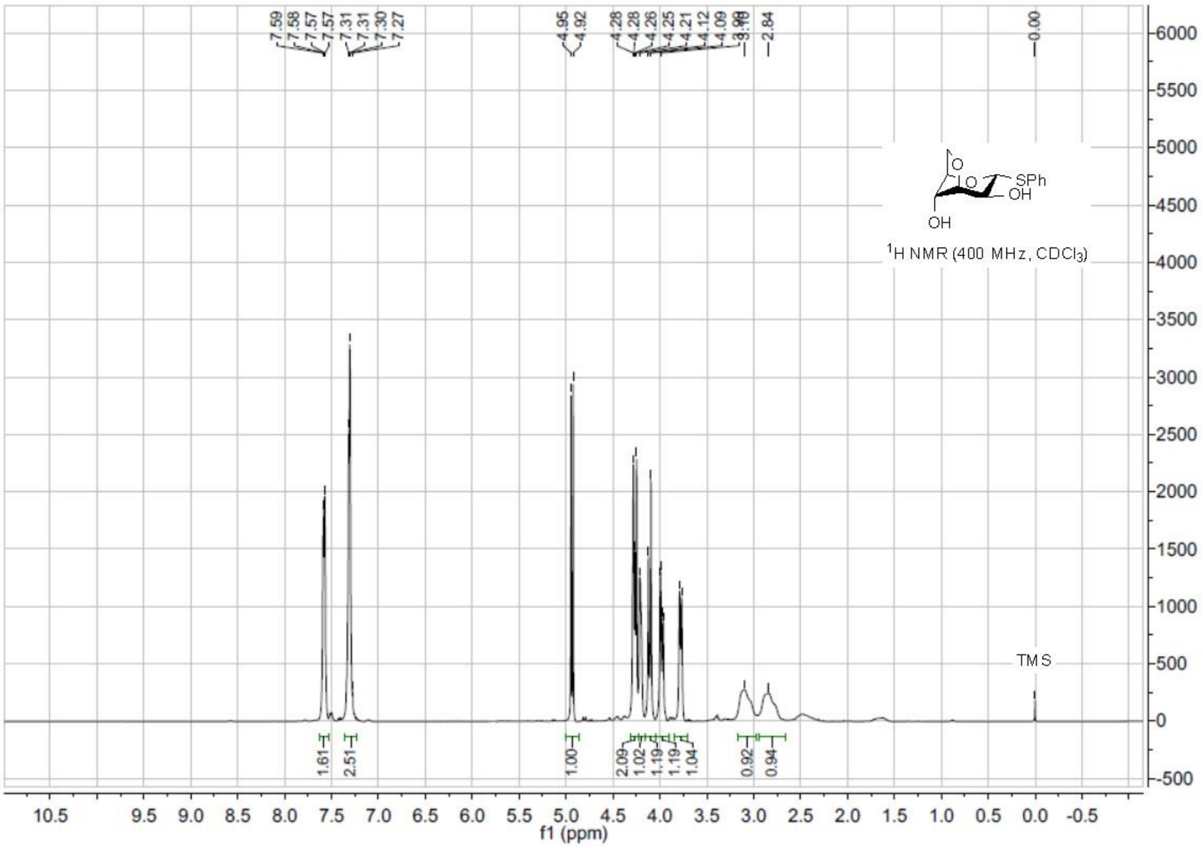

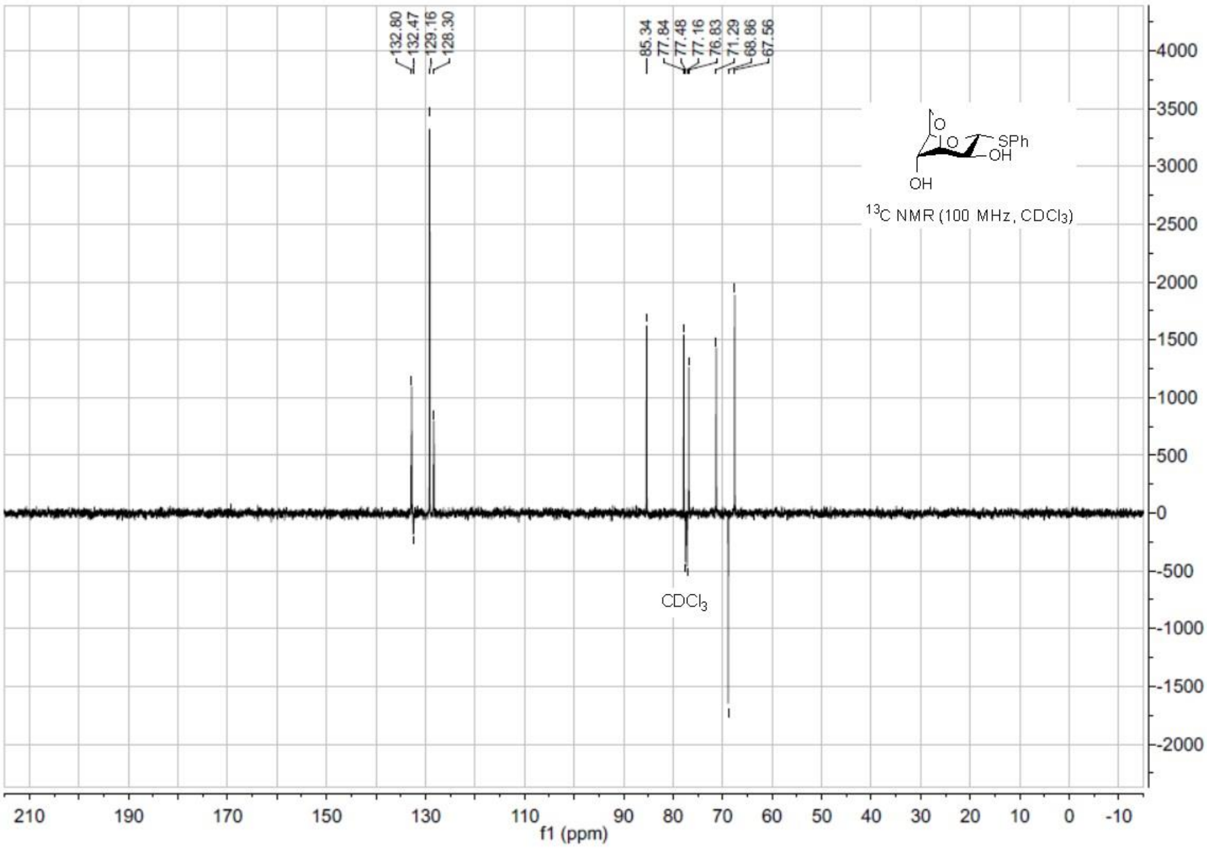

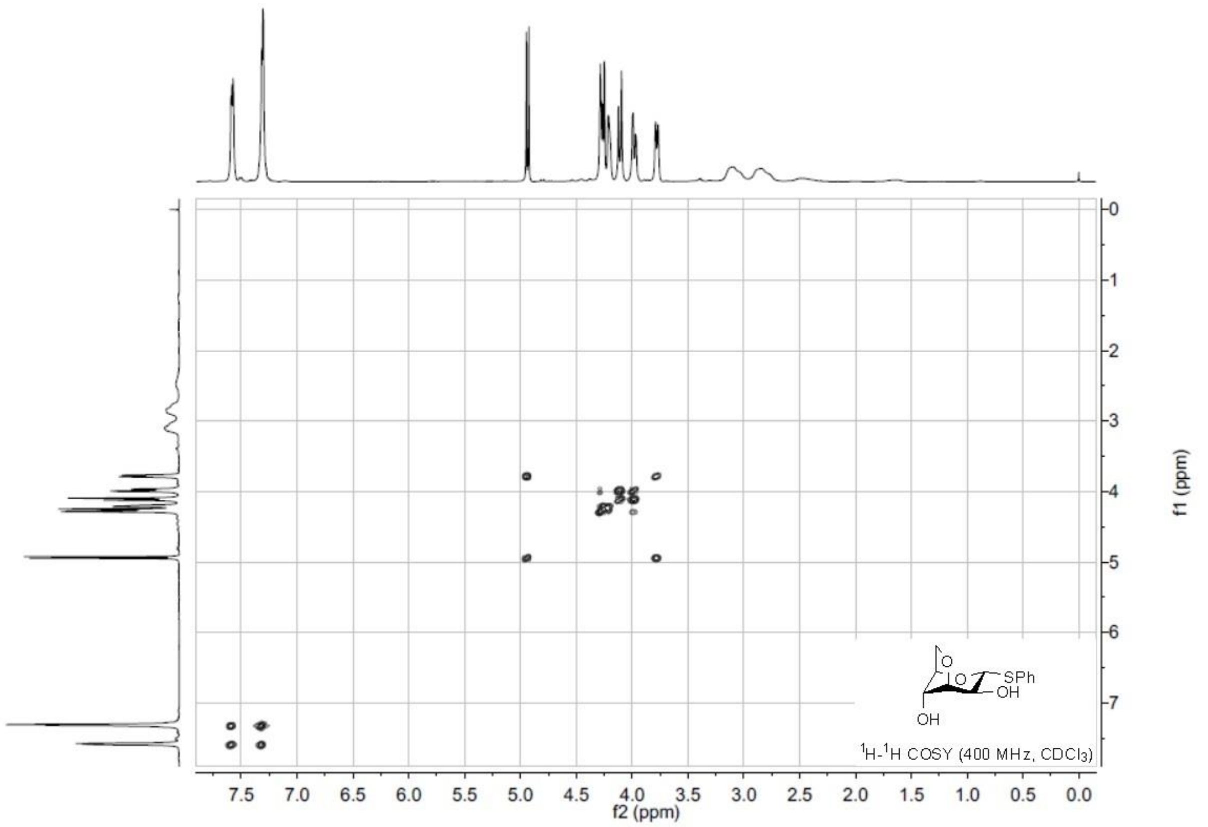
SPh
OH
OH
¹H-¹H COSY (400 MHz, CDCl3)
f2 (ppm)
f1 (ppm)

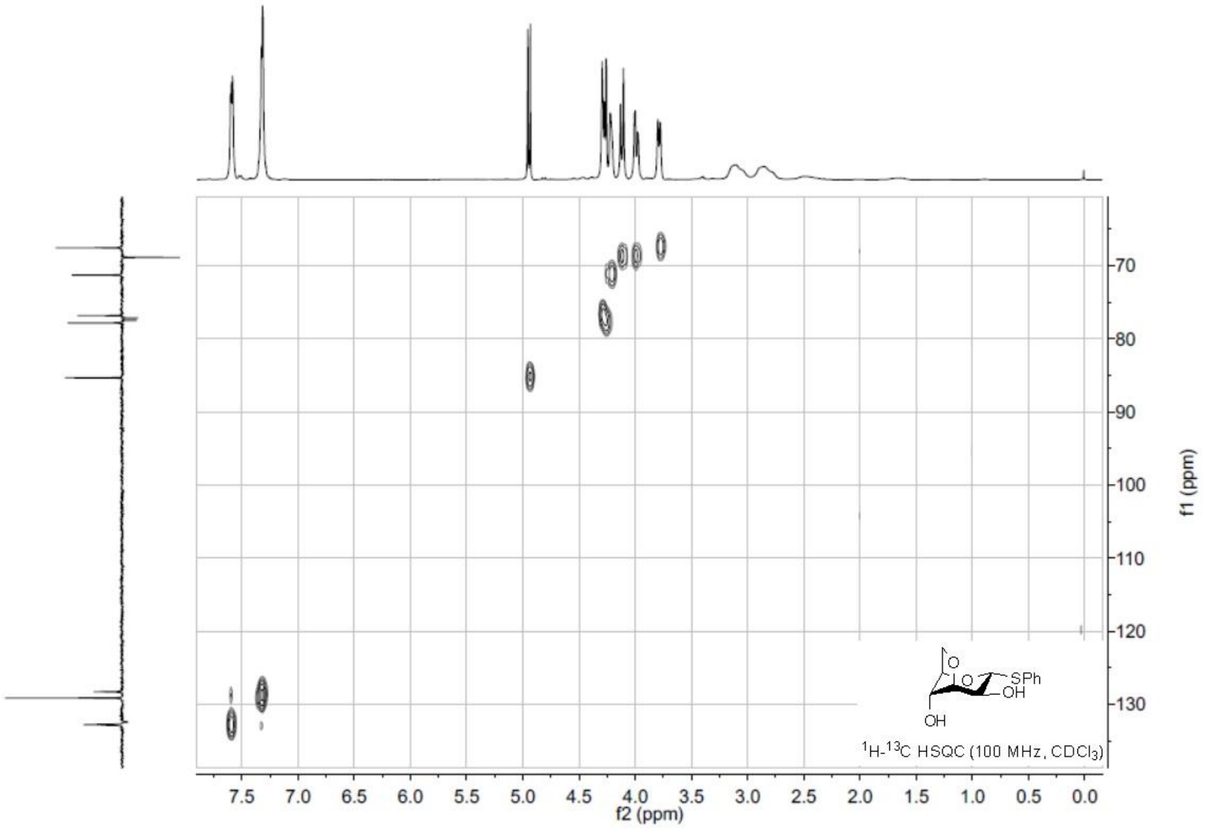
SPh
OH
OH
¹H-¹³C HSQC (100 MHz, CDCl3)
f2 (ppm)
f1 (ppm)

^{1}H and ^{13}C NMR spectra of compound **45**

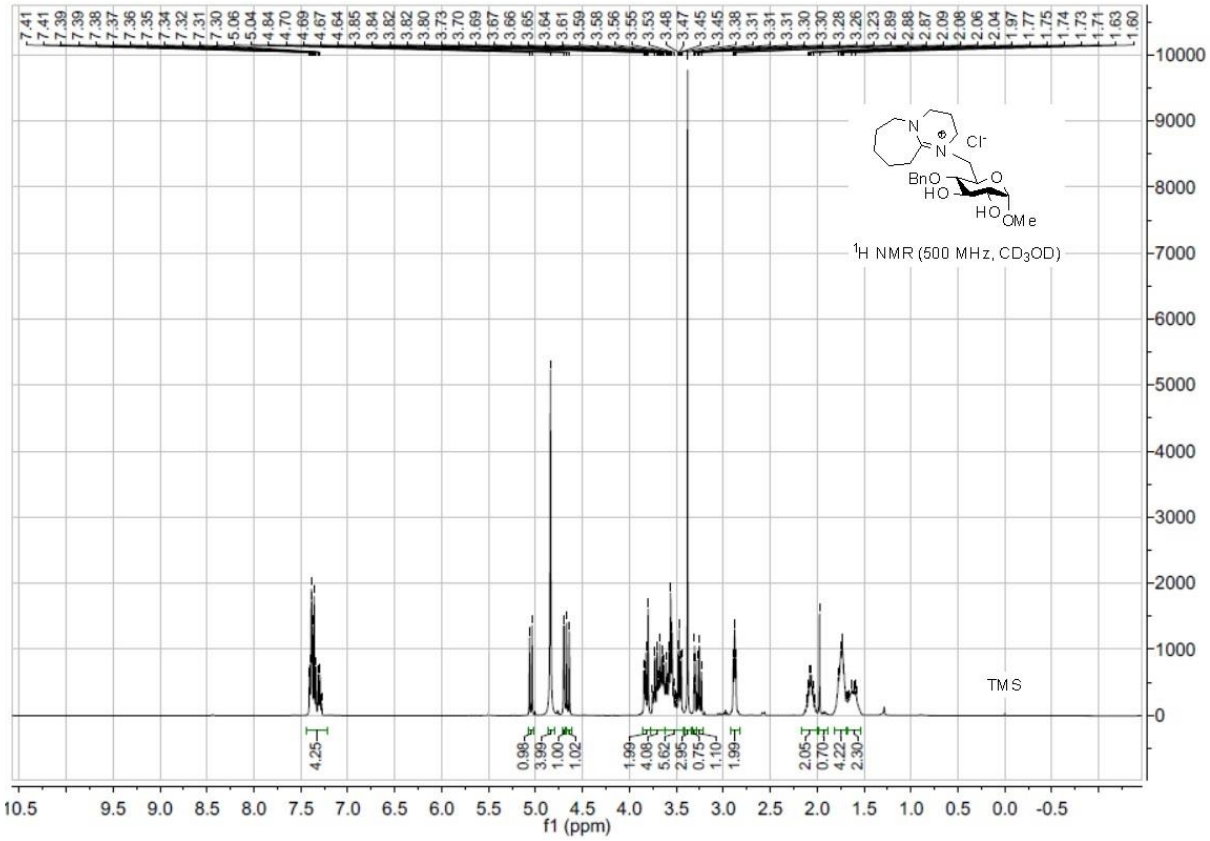

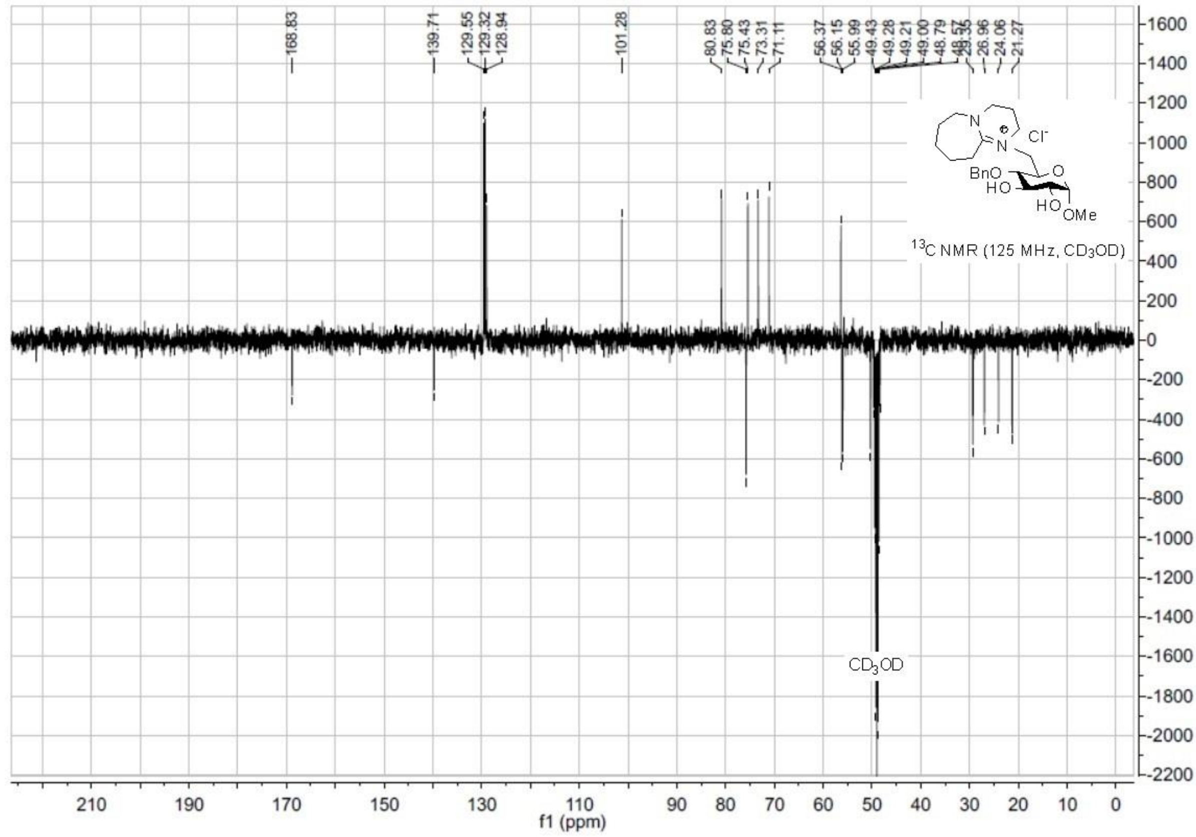

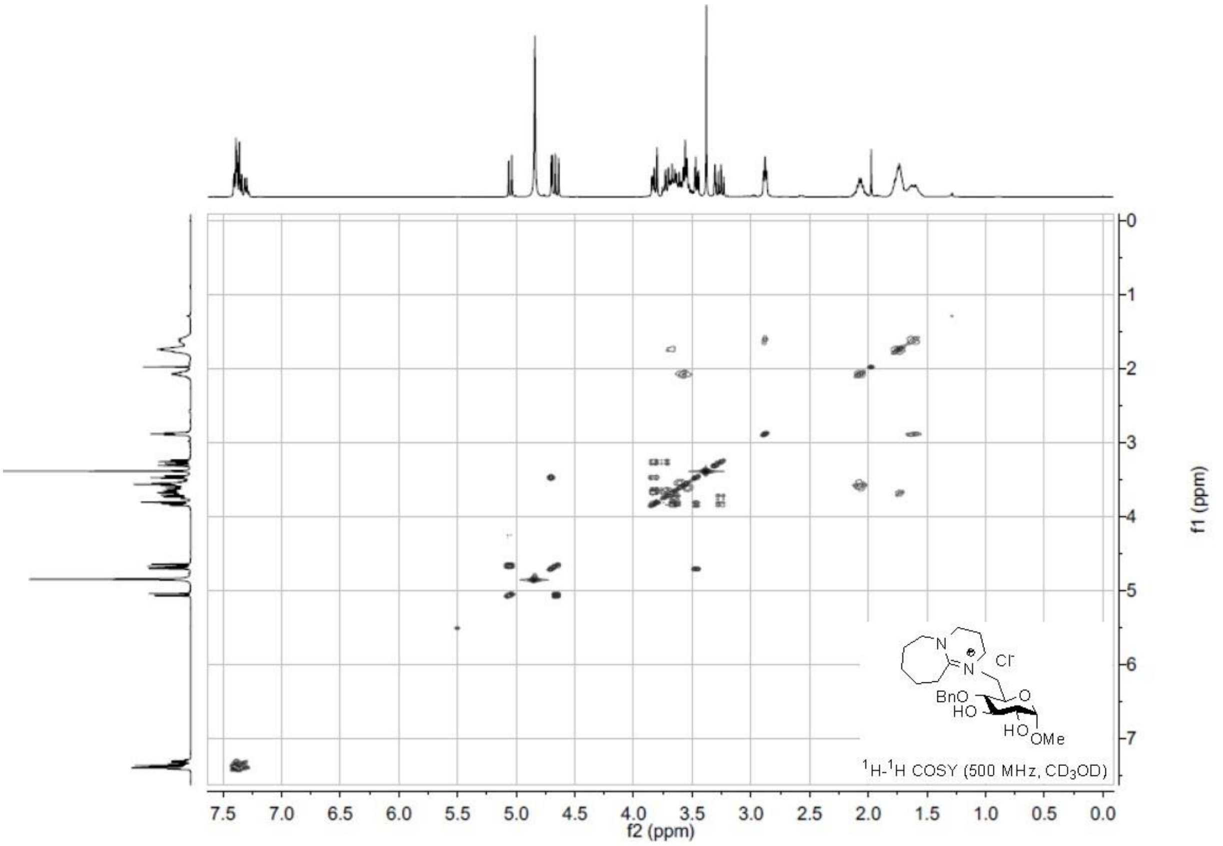

f2 (ppm)
f1 (ppm)
BnO
HO
HO
OMe
Cl⁻
^{1}H-^{1}H COSY (500 MHz, CD$_3$OD)

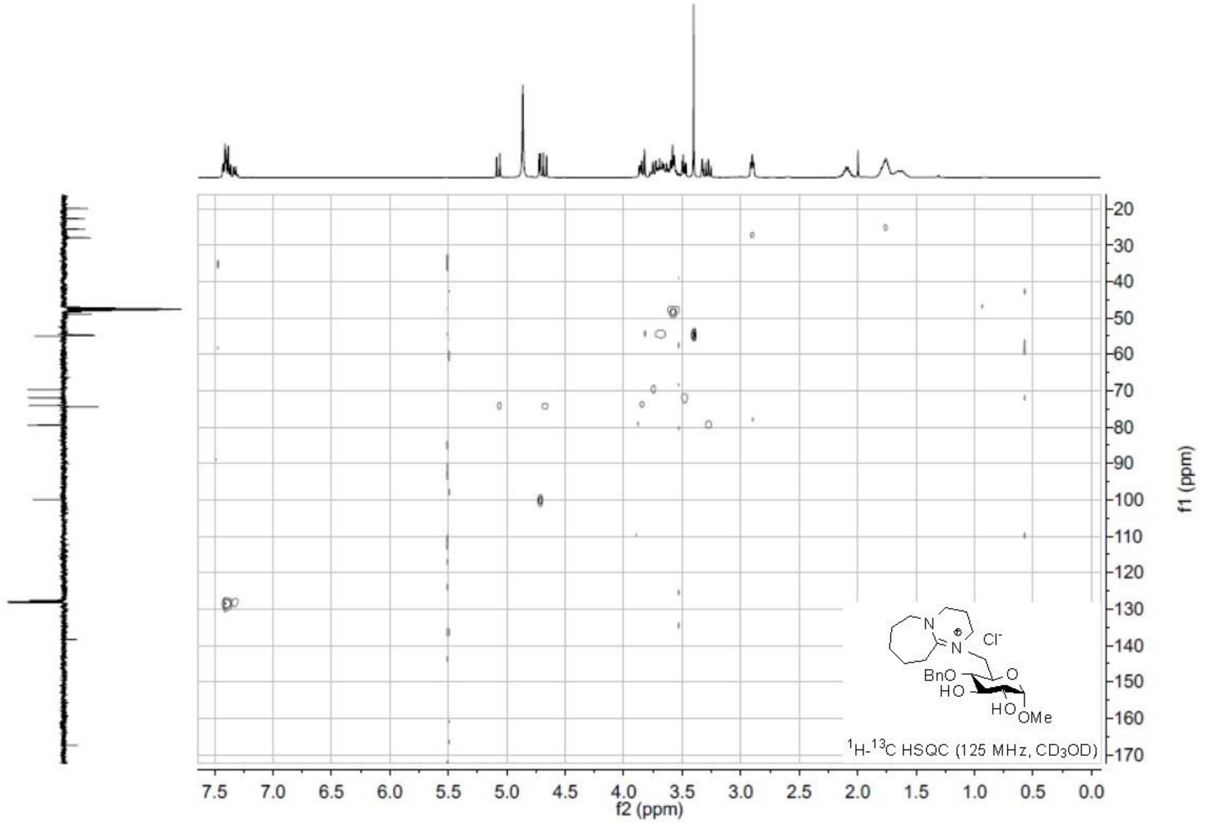

f2 (ppm)
f1 (ppm)
BnO
HO
HO
OMe
Cl⁻
^{1}H-^{13}C HSQC (125 MHz, CD$_3$OD)

^{1}H and ^{13}C NMR spectra of compound **46**

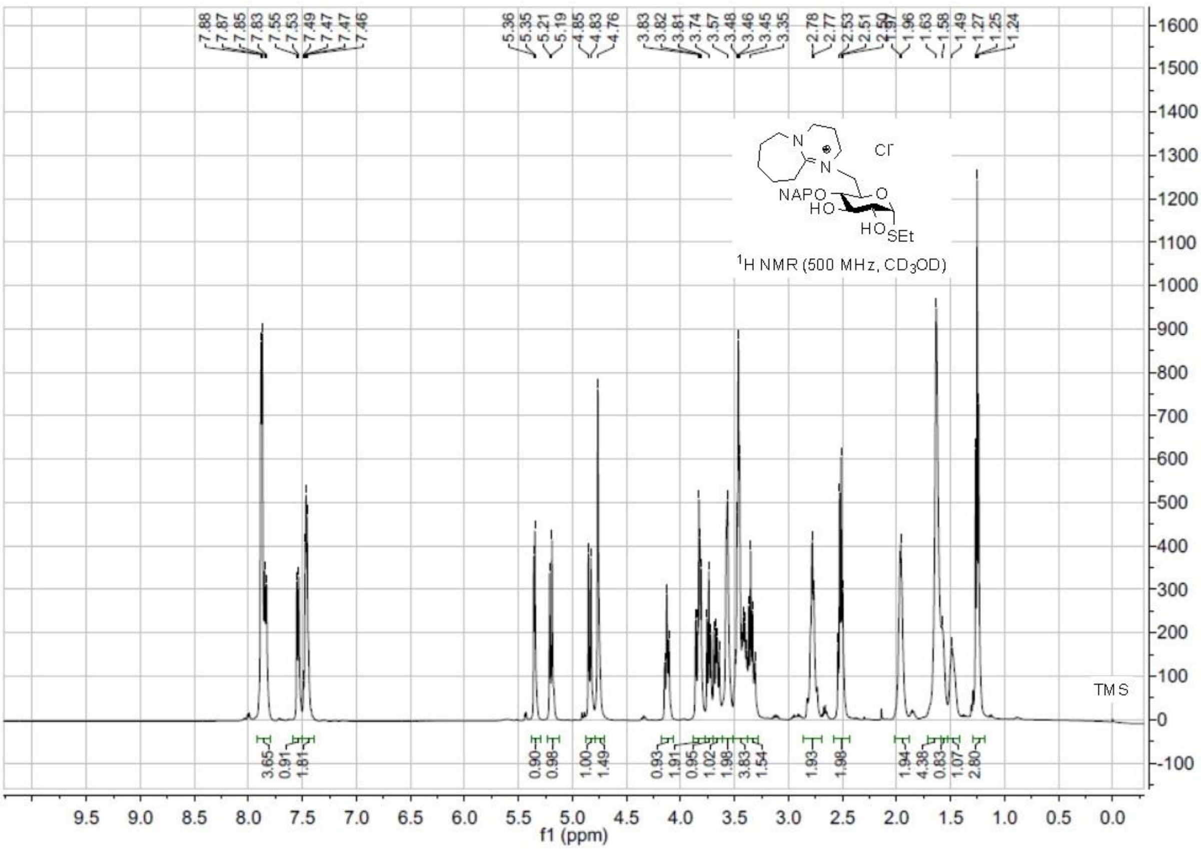

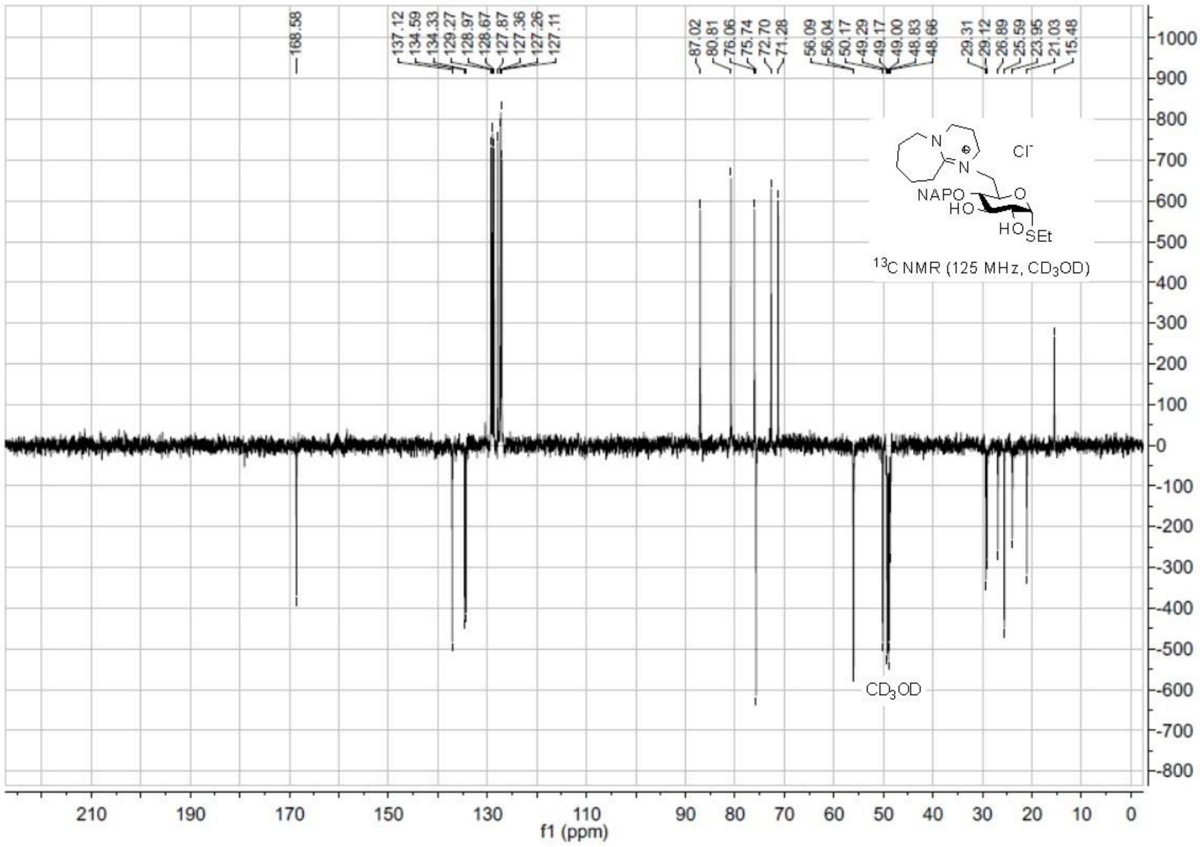

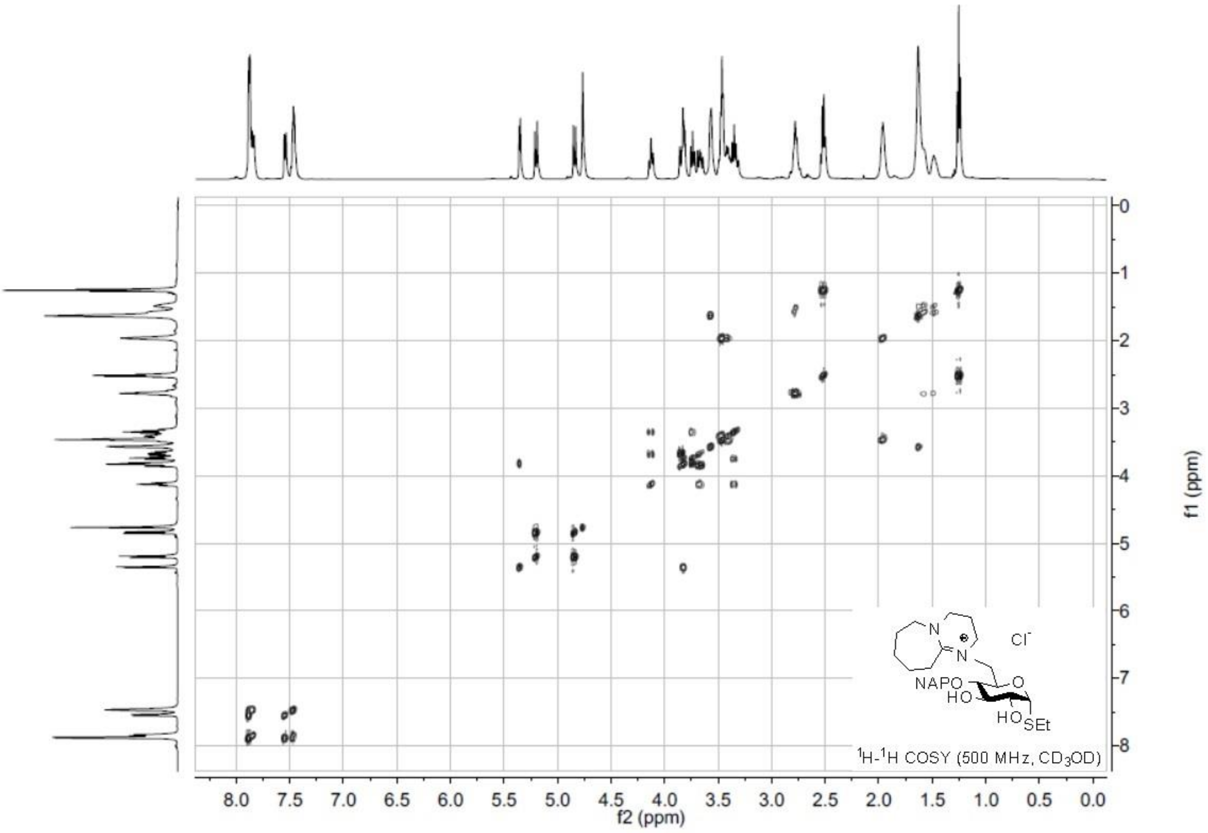
Cl-
NAPO
HO
HO
SEt
^{1}H-^{1}H COSY (500 MHz, CD$_3$OD)
f2 (ppm)
f1 (ppm)

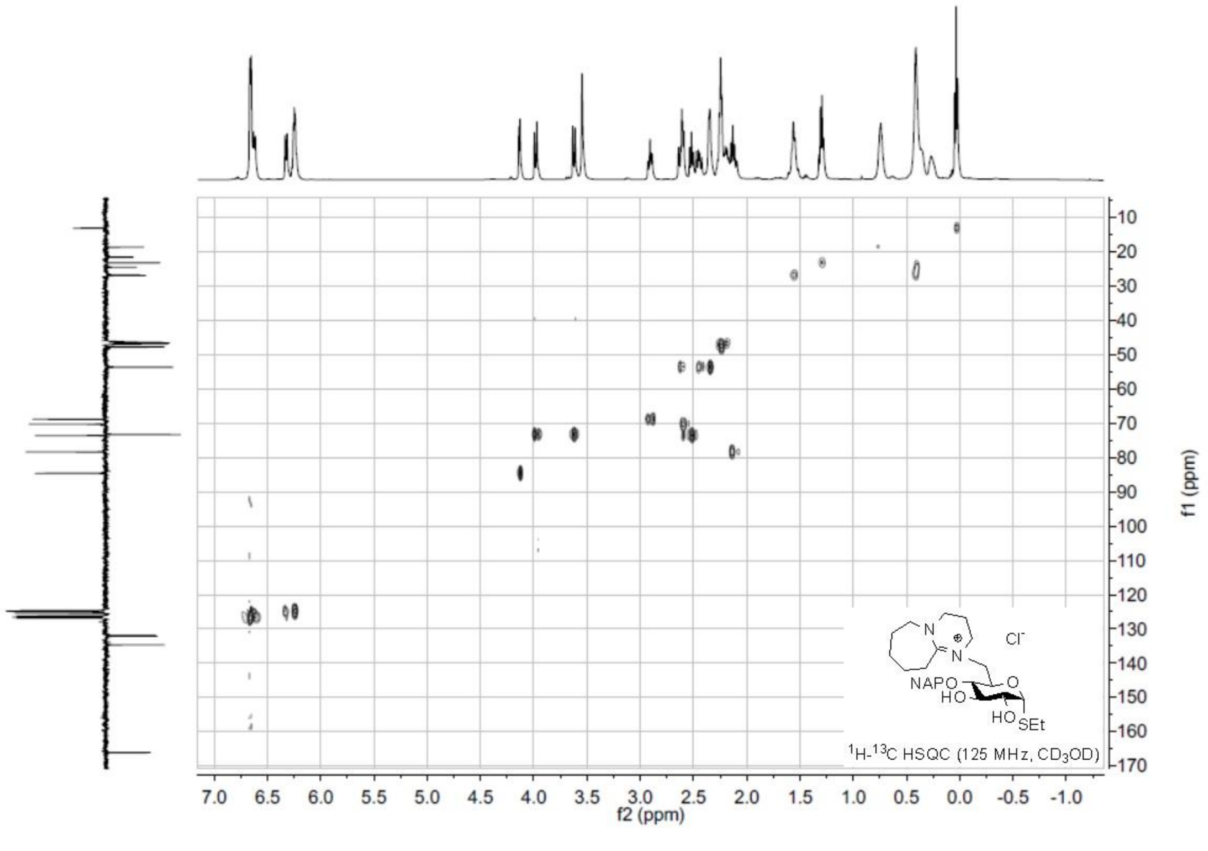
Cl-
NAPO
HO
HO
SEt
^{1}H-^{13}C HSQC (125 MHz, CD$_3$OD)
f2 (ppm)
f1 (ppm)

^{1}H and ^{13}C NMR spectra of compound **47**

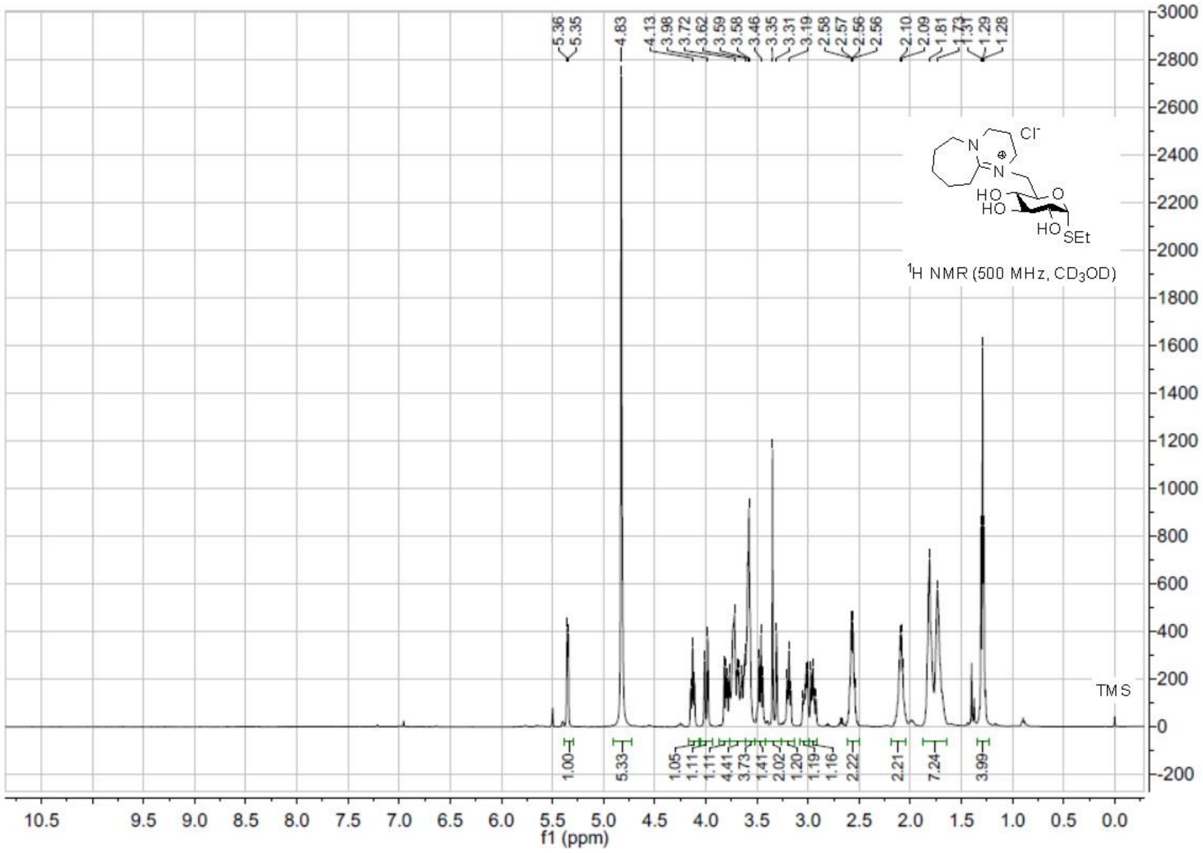

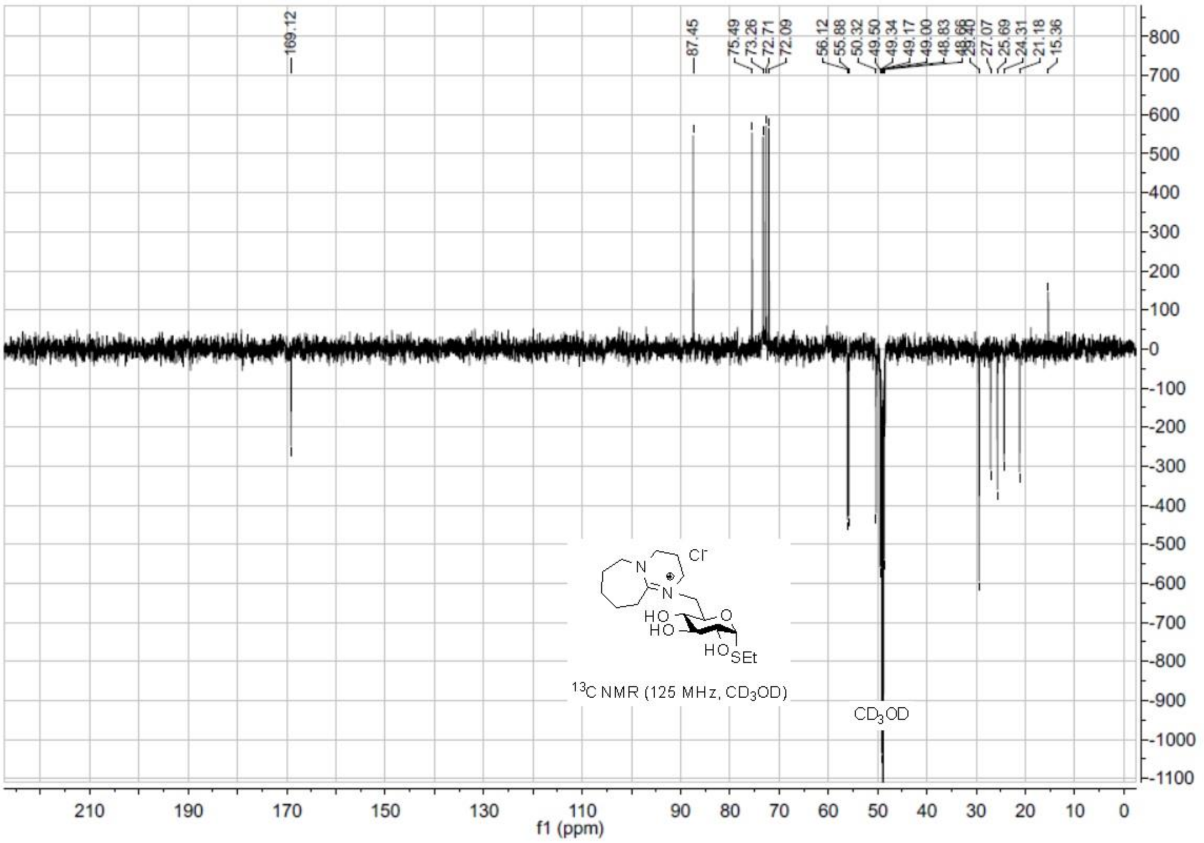

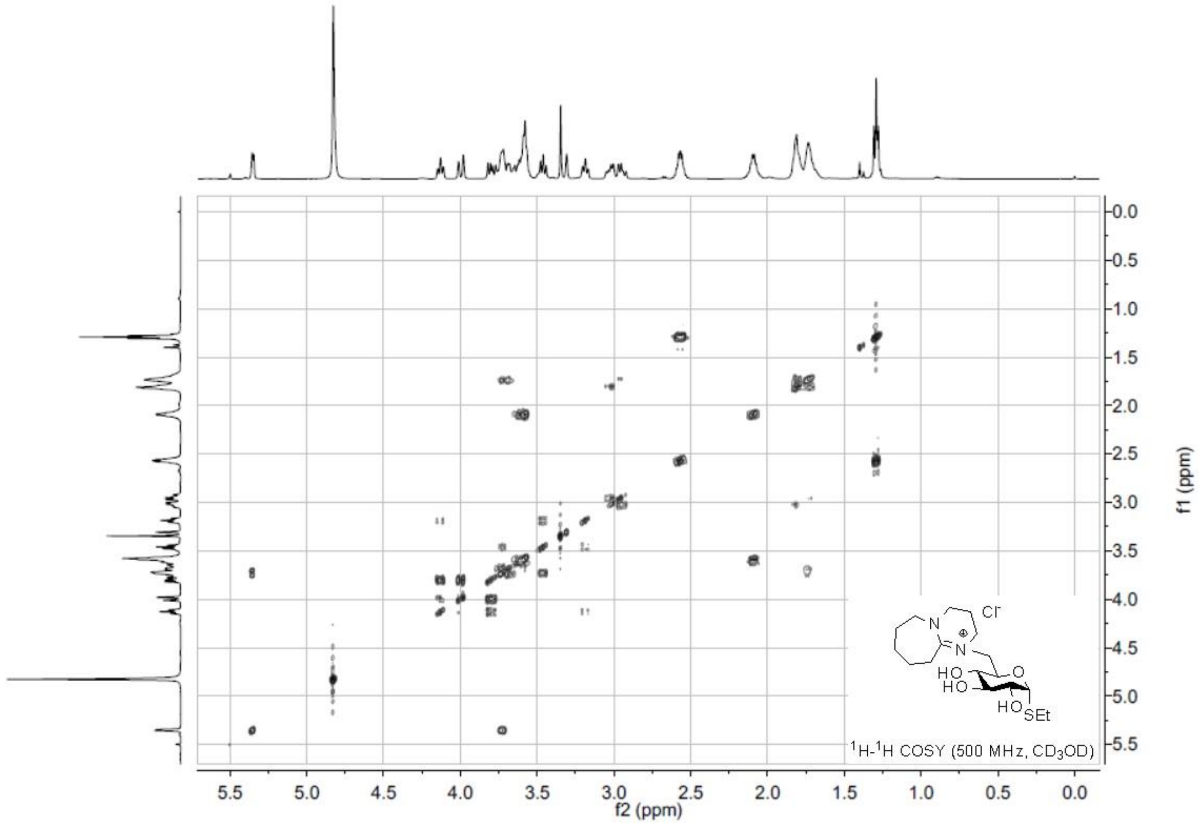

Cl-
N
N
HO
HO
HO
O
SEt
^{1}H-^{1}H COSY (500 MHz, CD$_3$OD)
f2 (ppm)
f1 (ppm)

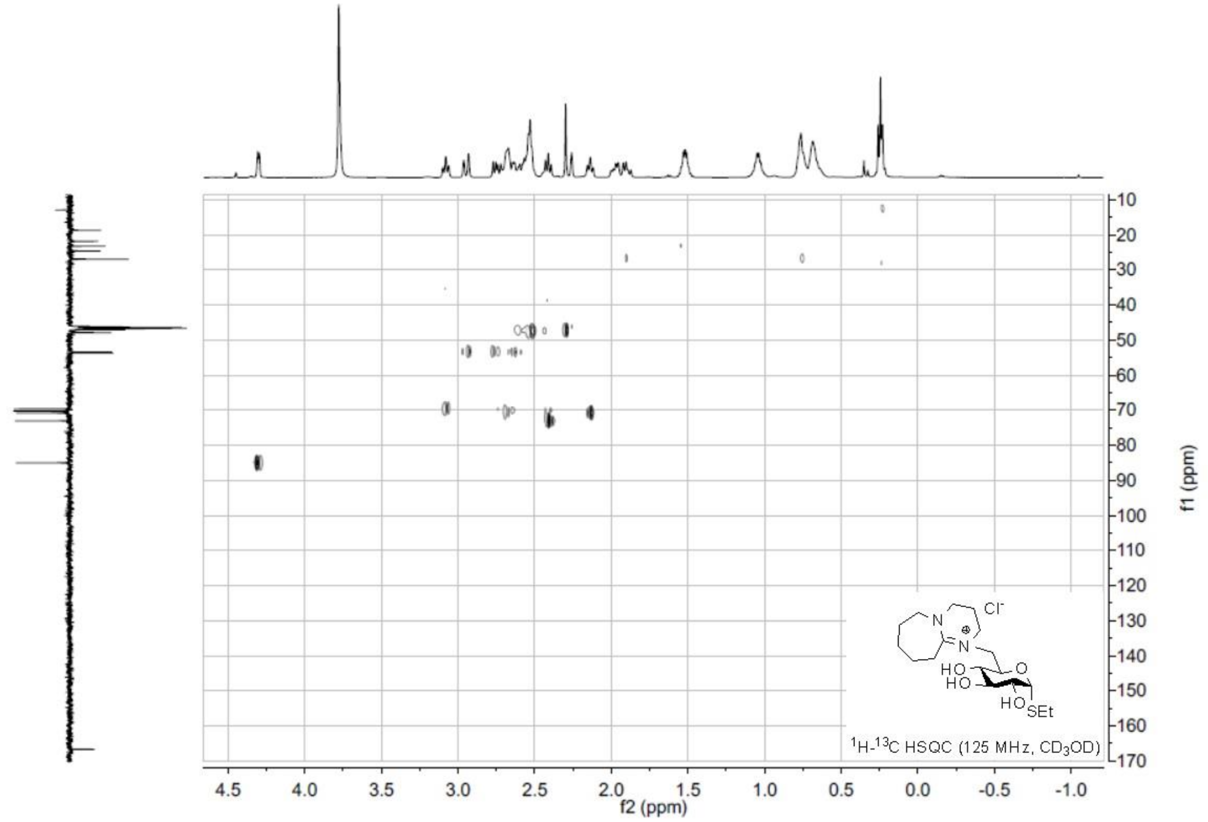

Cl-
N
N
HO
HO
HO
O
SEt
^{1}H-^{13}C HSQC (125 MHz, CD$_3$OD)
f2 (ppm)
f1 (ppm)

^{1}H and ^{13}C NMR spectra of compound **48**

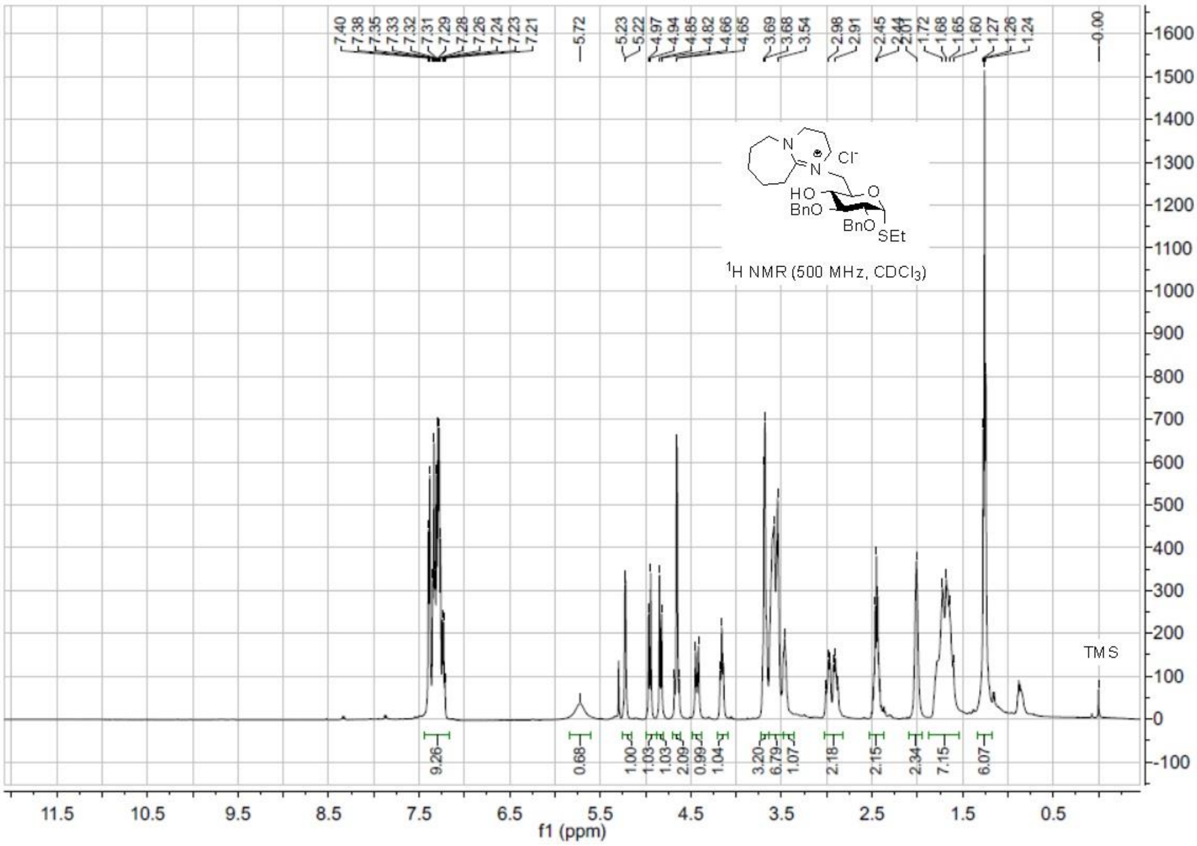

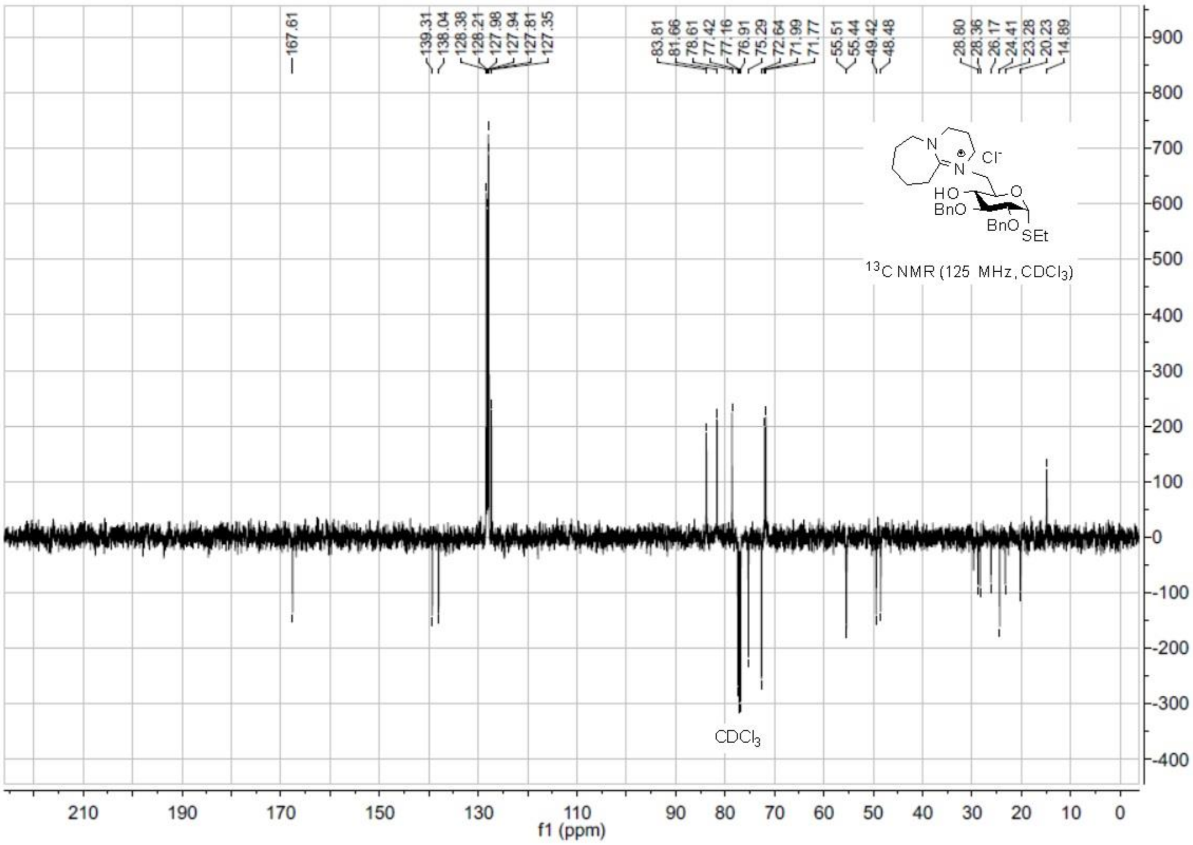

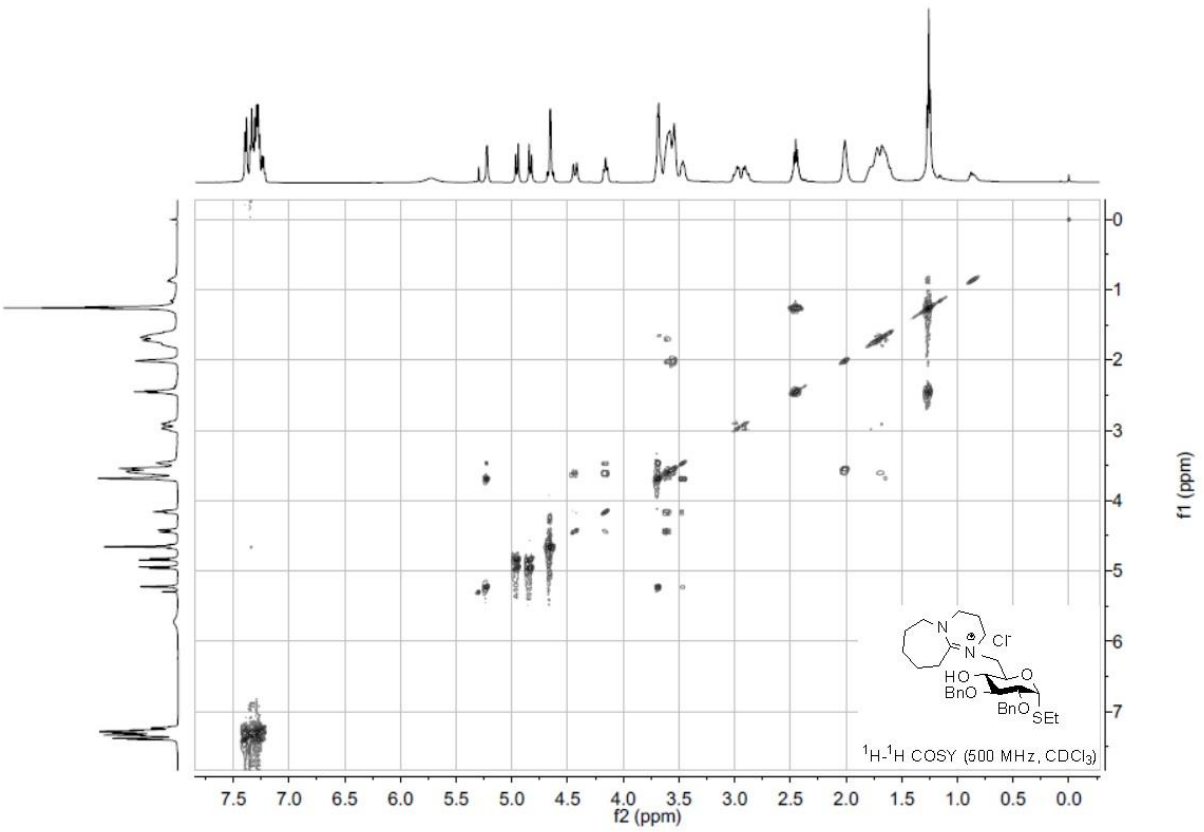
Cl⁻
HO
BnO
BnO
SEt
¹H-¹H COSY (500 MHz, CDCl₃)
f2 (ppm)
f1 (ppm)

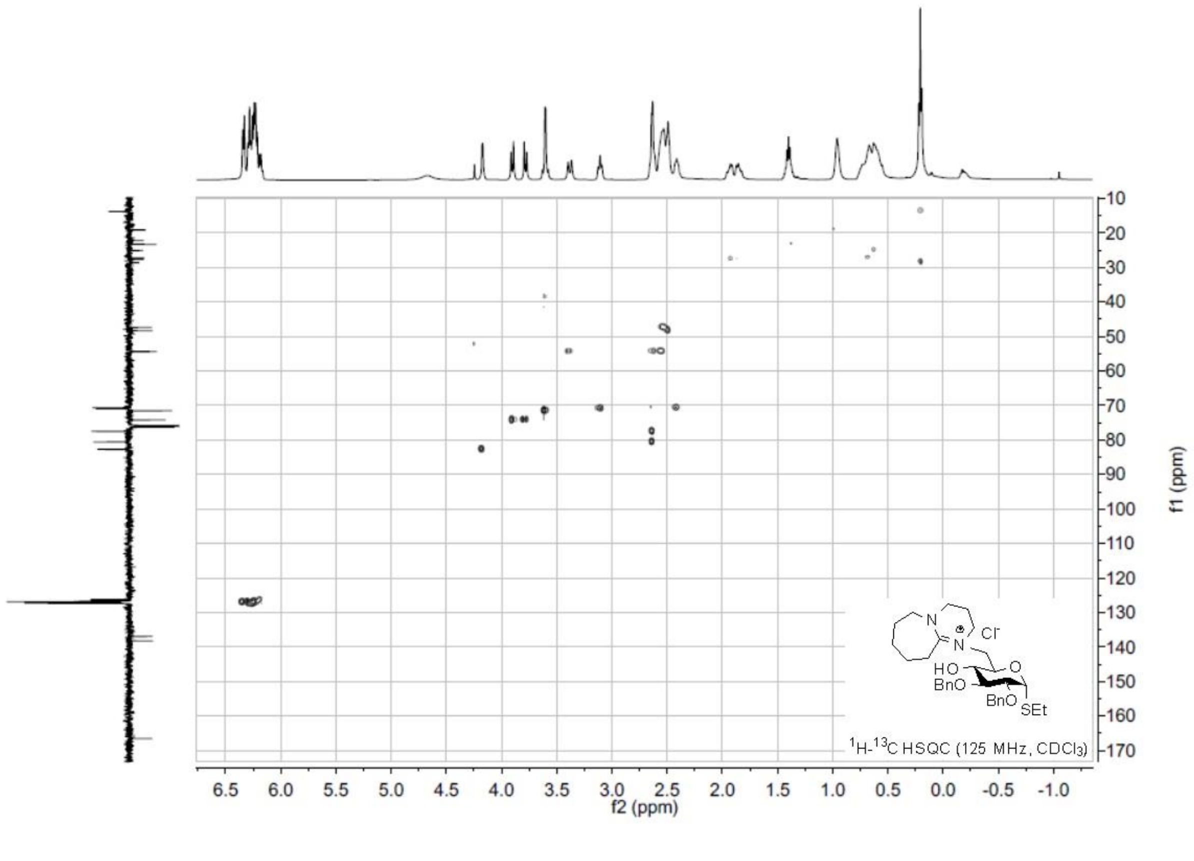
Cl⁻
HO
BnO
BnO
SEt
¹H-¹³C HSQC (125 MHz, CDCl₃)
f2 (ppm)
f1 (ppm)

^{1}H and ^{13}C NMR spectra of compound **49**

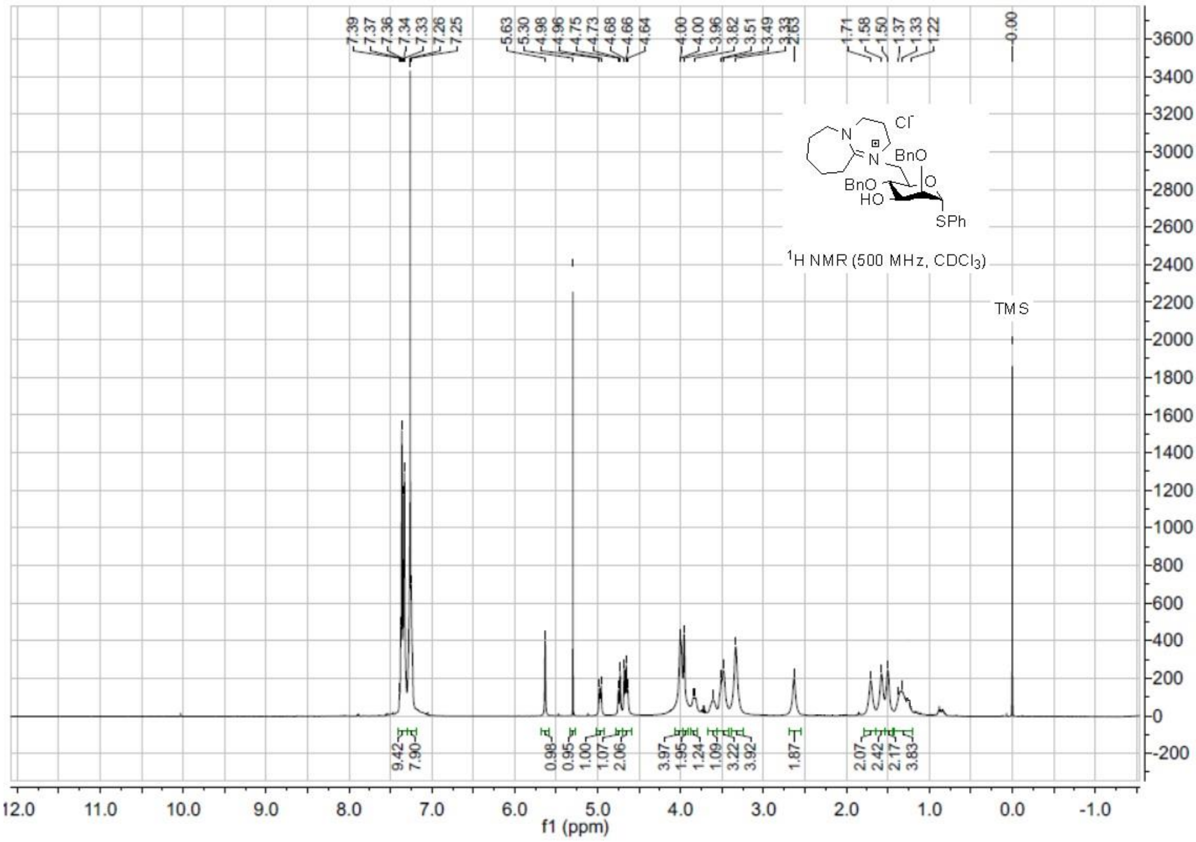

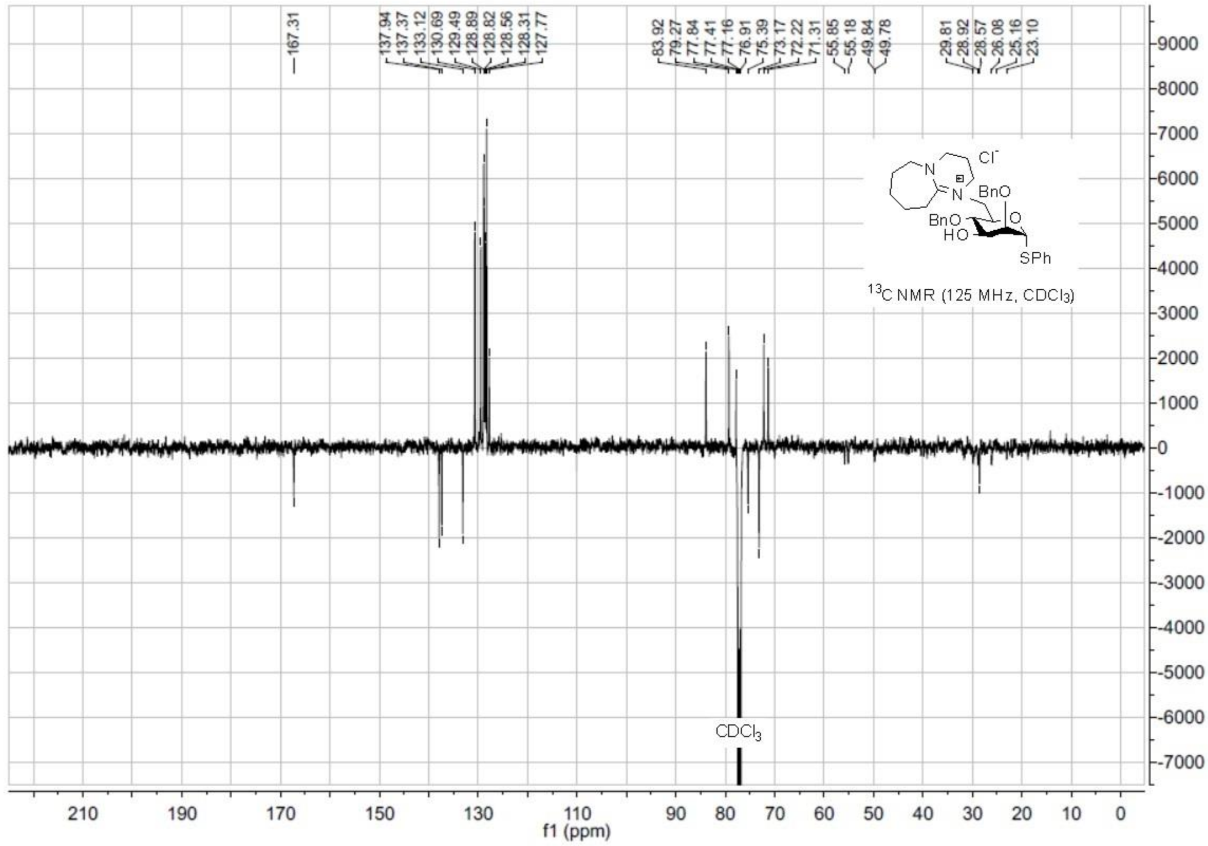

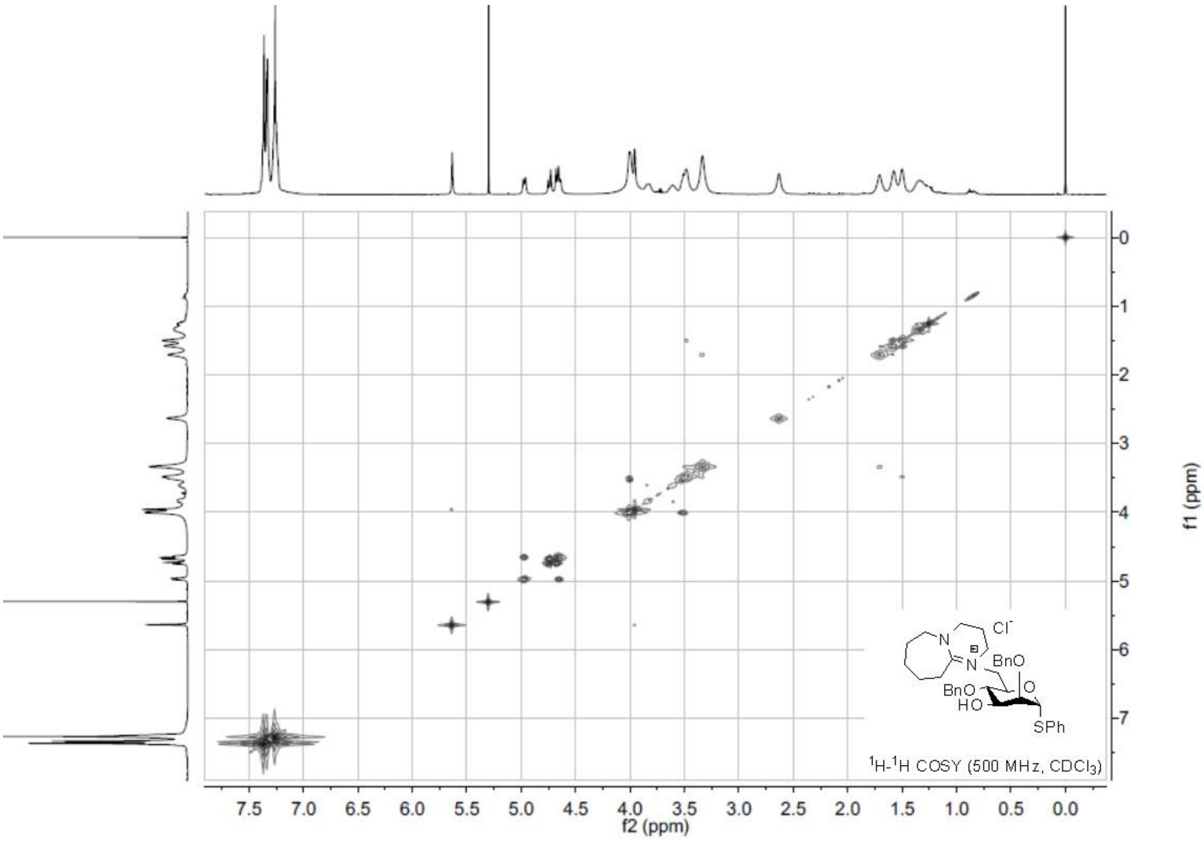
Cl⁻
N
N
BnO
BnO
O
HO
SPh
1H-1H COSY (500 MHz, CDCl3)
f1 (ppm)
f2 (ppm)

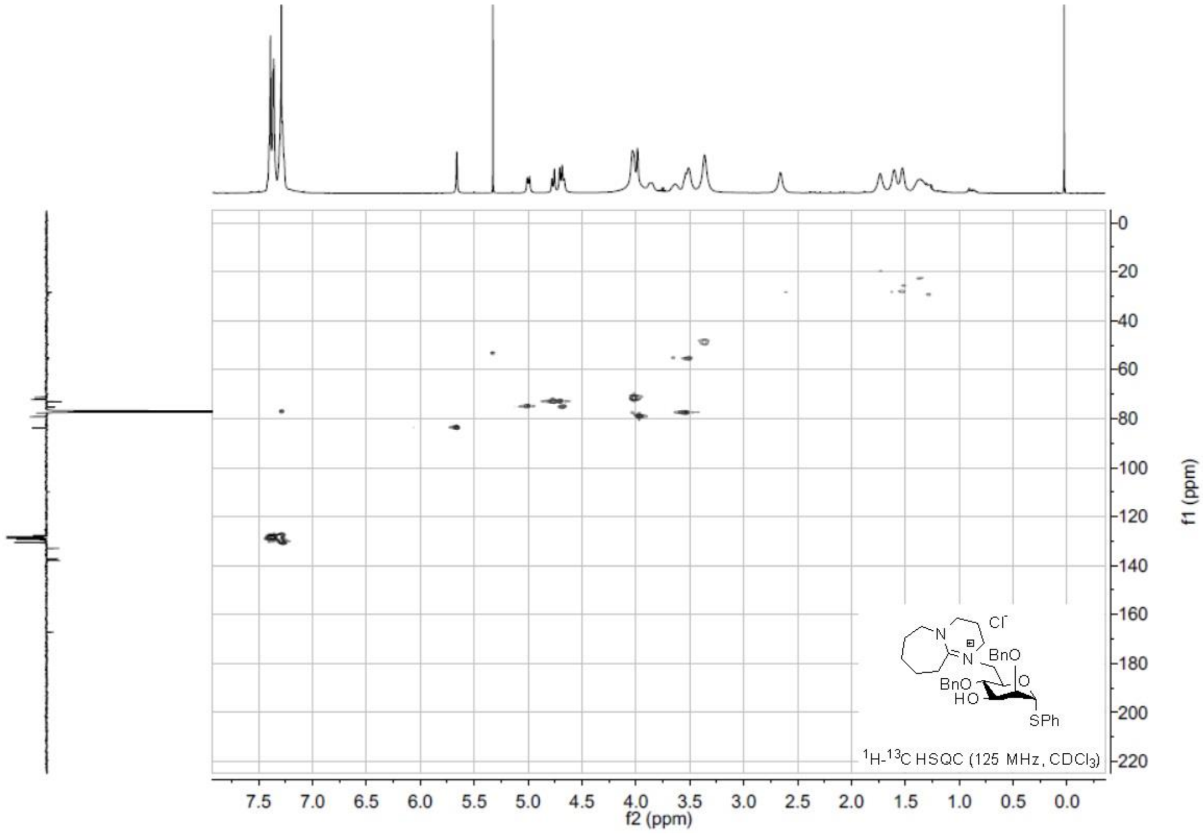
Cl⁻
N
N
BnO
BnO
O
HO
SPh
1H-13C HSQC (125 MHz, CDCl3)
f1 (ppm)
f2 (ppm)

^{1}H and ^{13}C NMR spectra of compound **50**

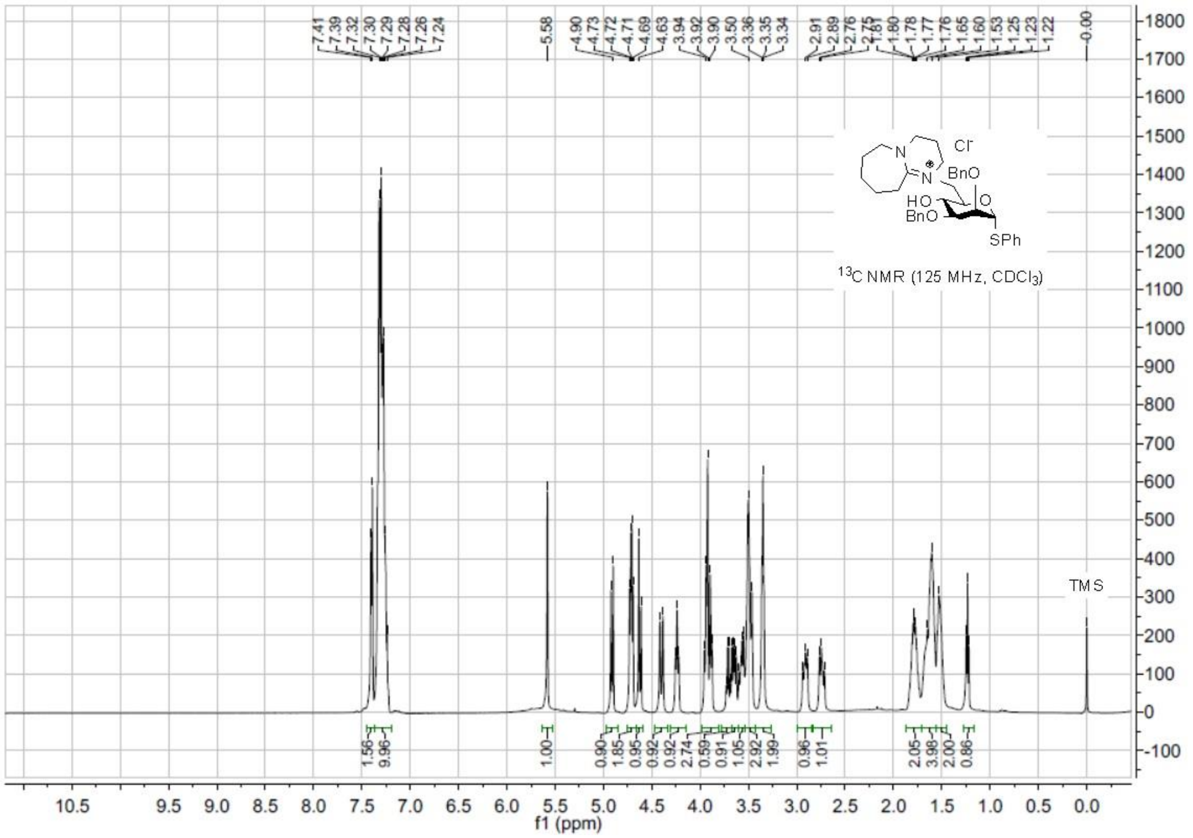

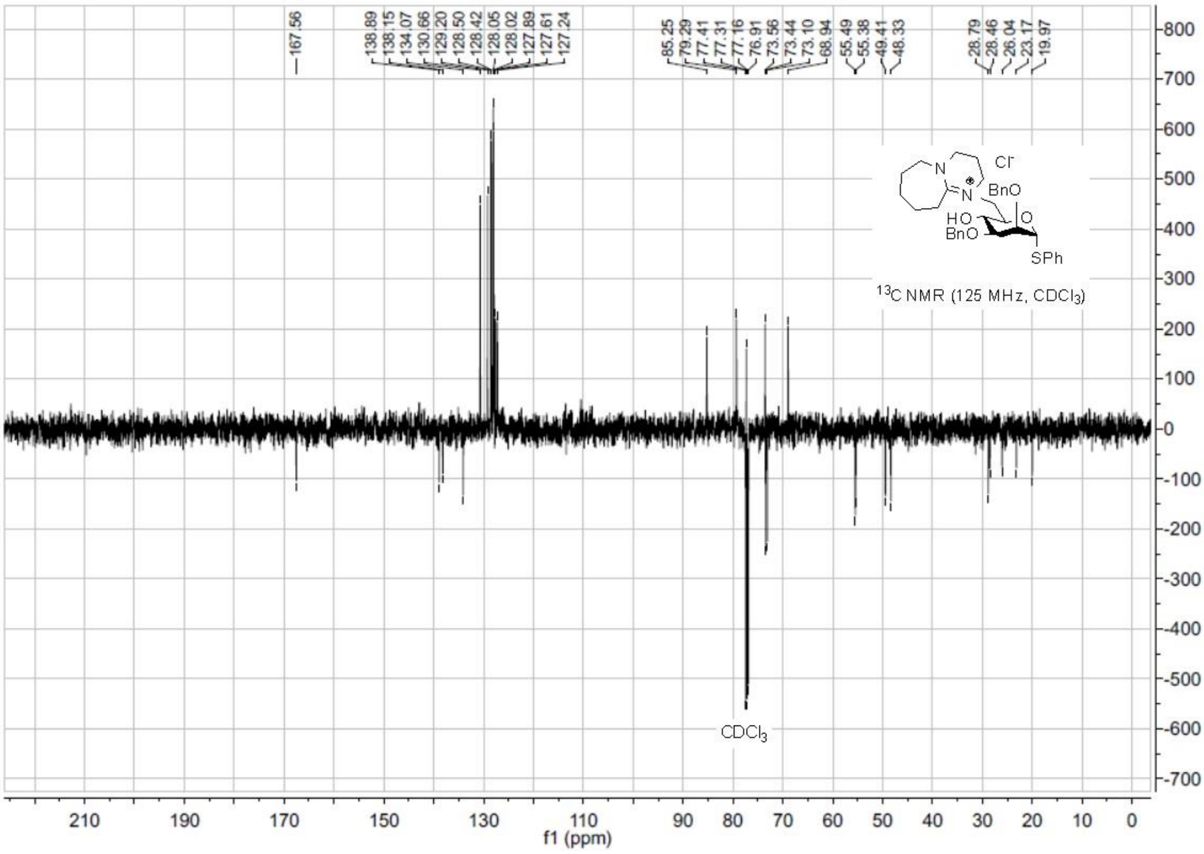

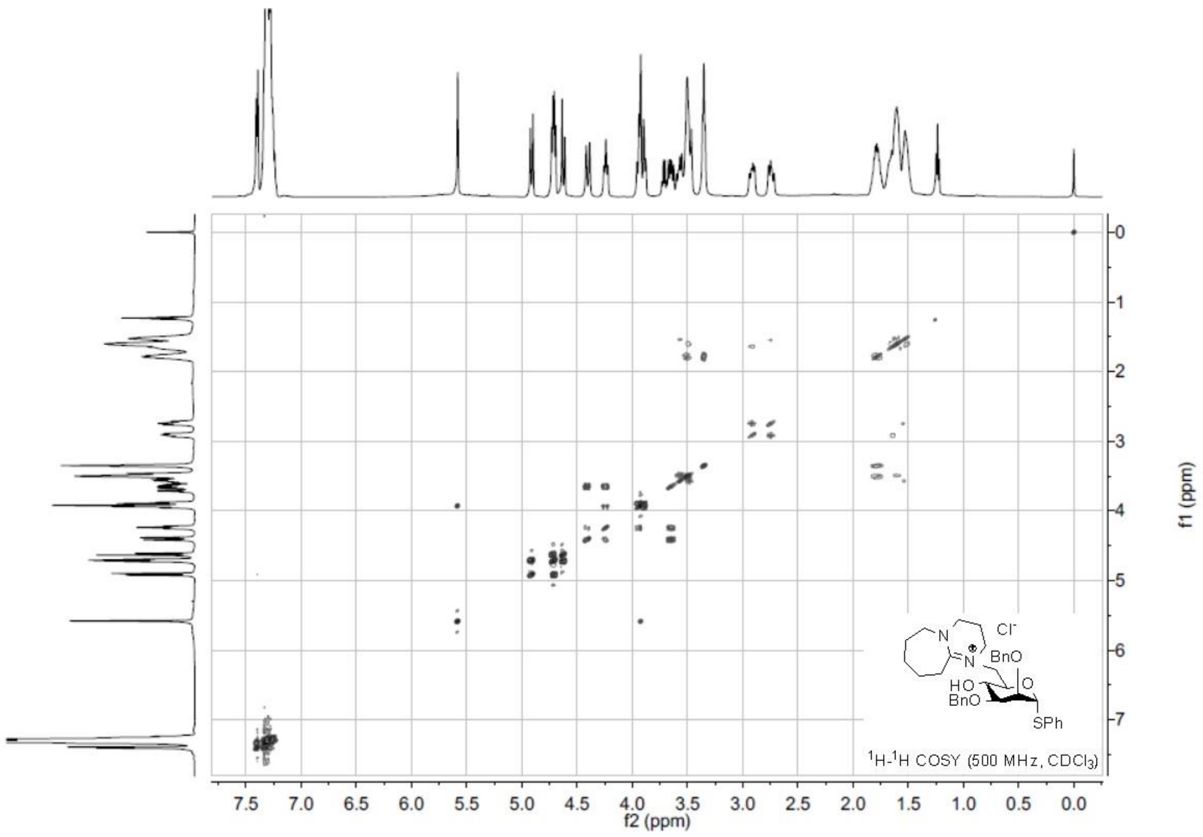
Cl⁻
BnO
HO
BnO
SPh
¹H-¹H COSY (500 MHz, CDCl₃)
f2 (ppm)
f1 (ppm)

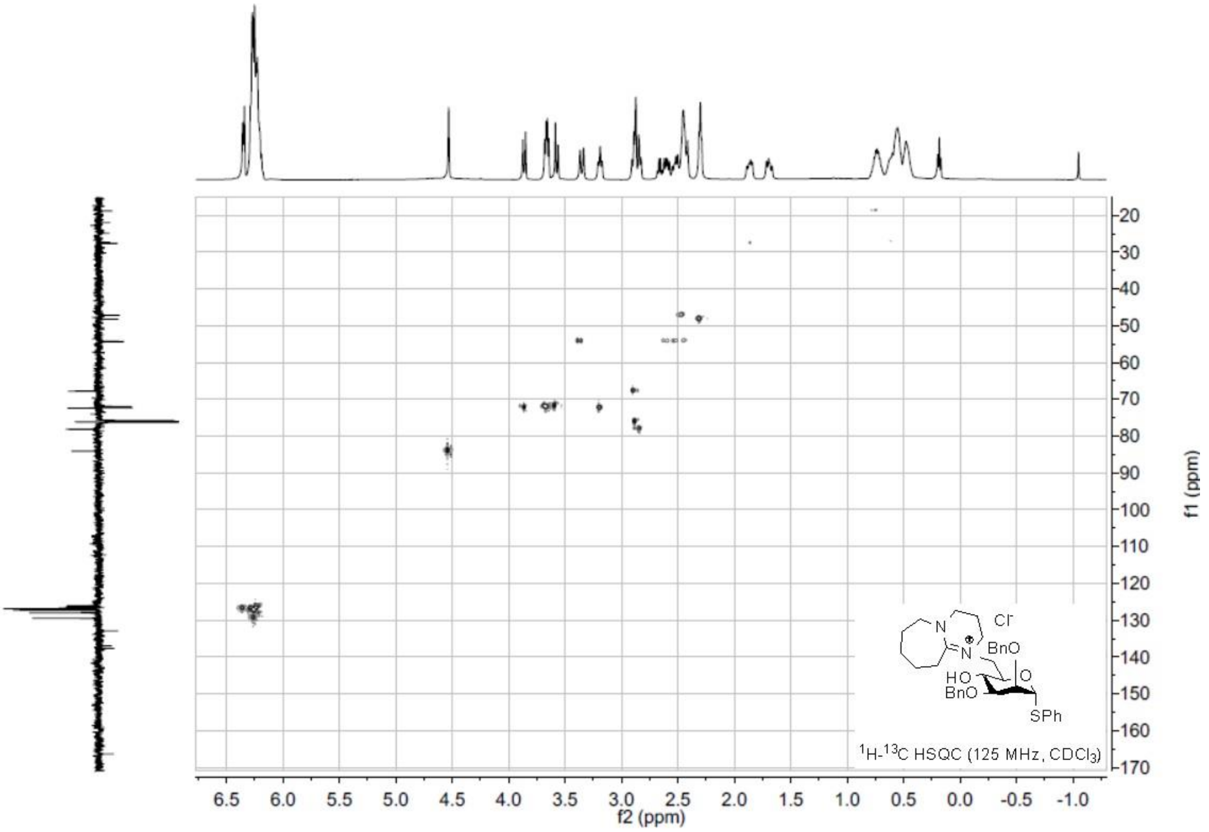
Cl⁻
BnO
HO
BnO
SPh
¹H-¹³C HSQC (125 MHz, CDCl₃)
f2 (ppm)
f1 (ppm)

^{1}H and ^{13}C NMR spectra of compound **51**

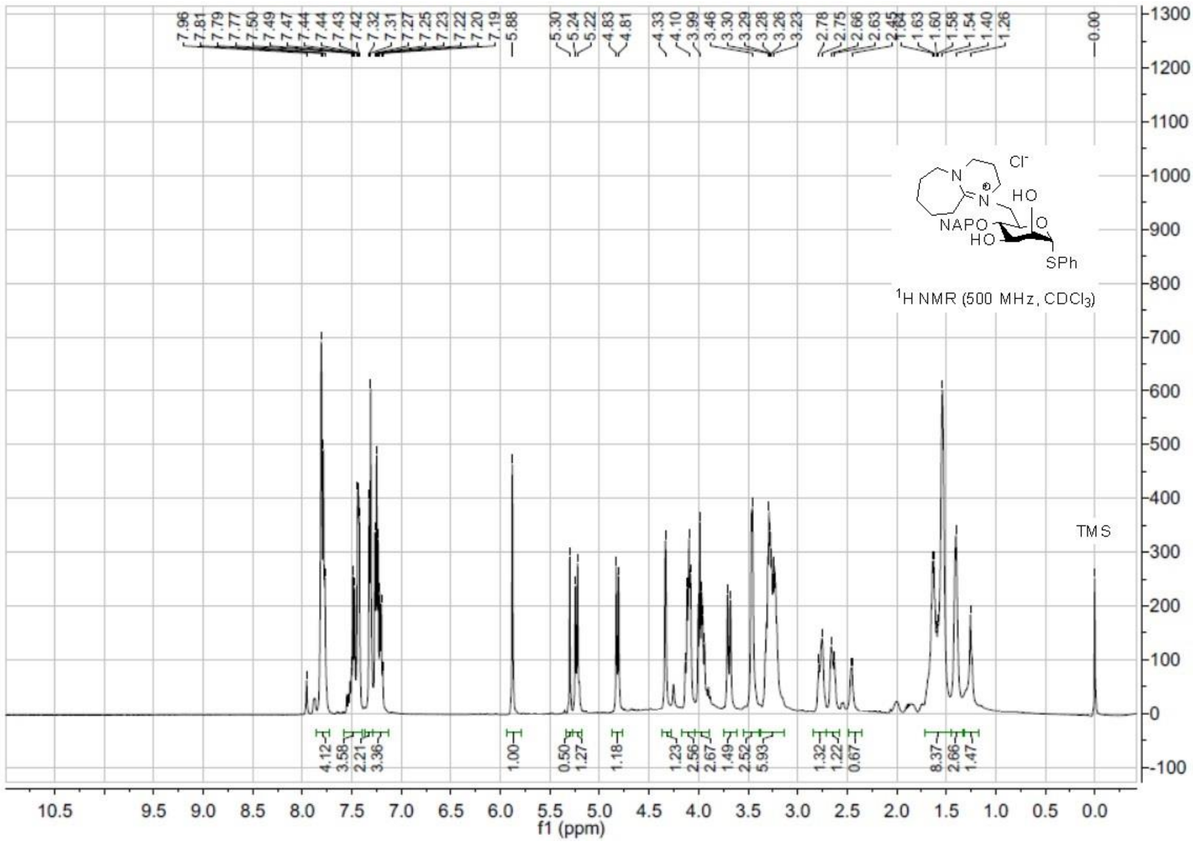

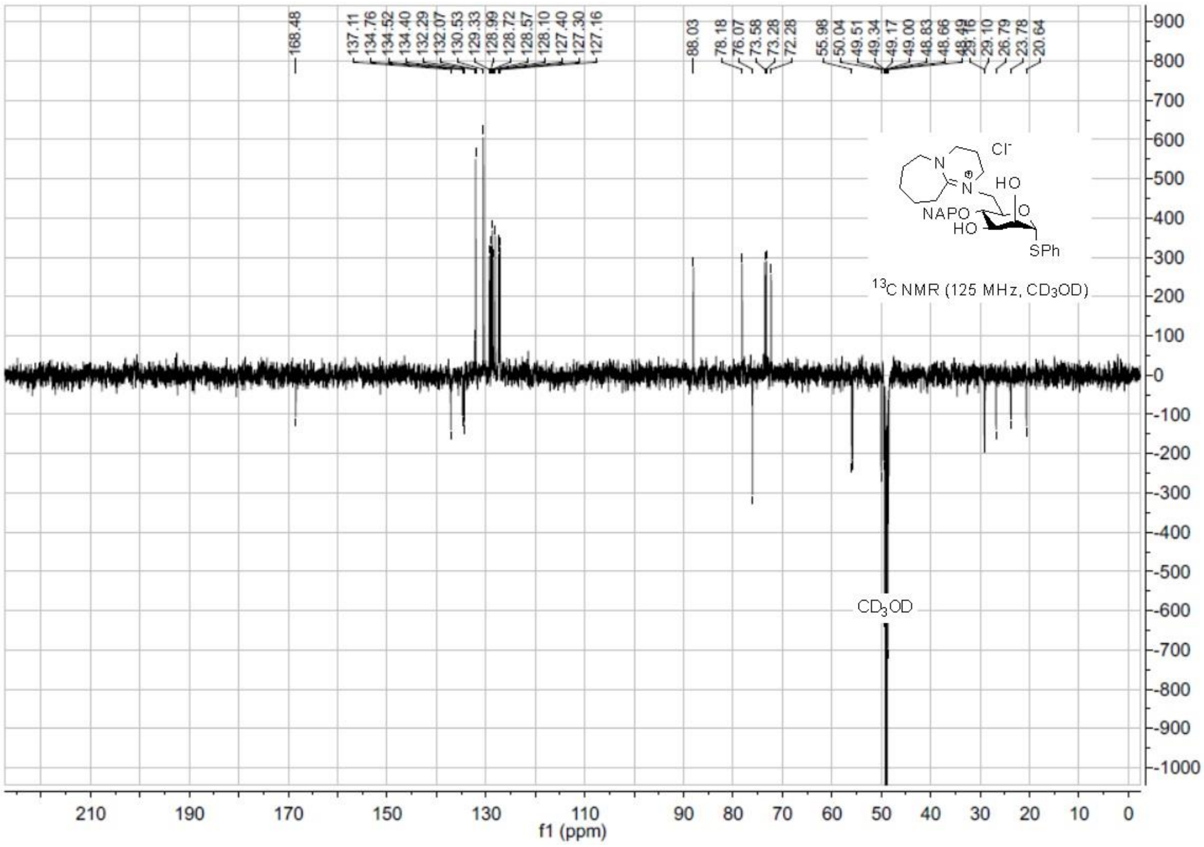

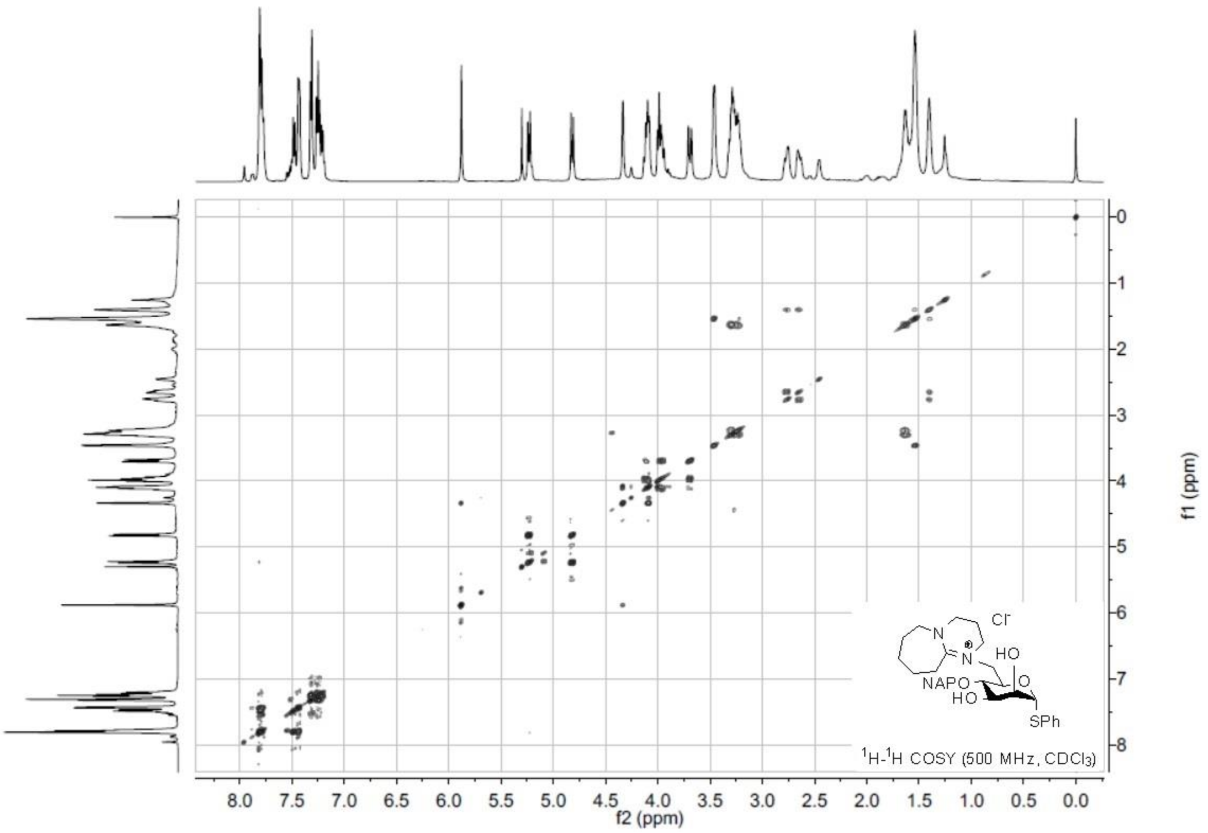
Cl⁻
N
N
HO
NAPO
HO
O
SPh
¹H-¹H COSY (500 MHz, CDCl₃)
f1 (ppm)
f2 (ppm)

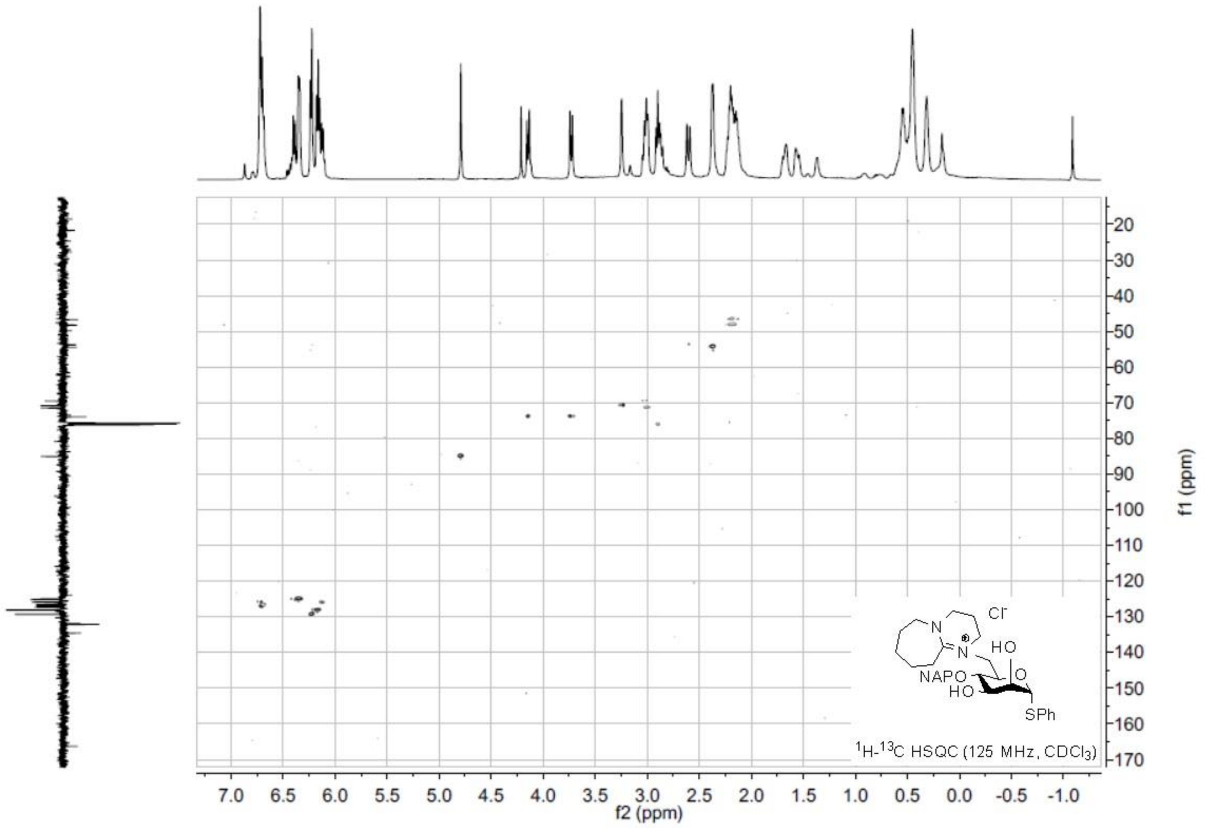
Cl⁻
N
N
HO
NAPO
HO
O
SPh
¹H-¹³C HSQC (125 MHz, CDCl₃)
f1 (ppm)
f2 (ppm)

^{1}H and ^{13}C NMR spectra of compound **52**

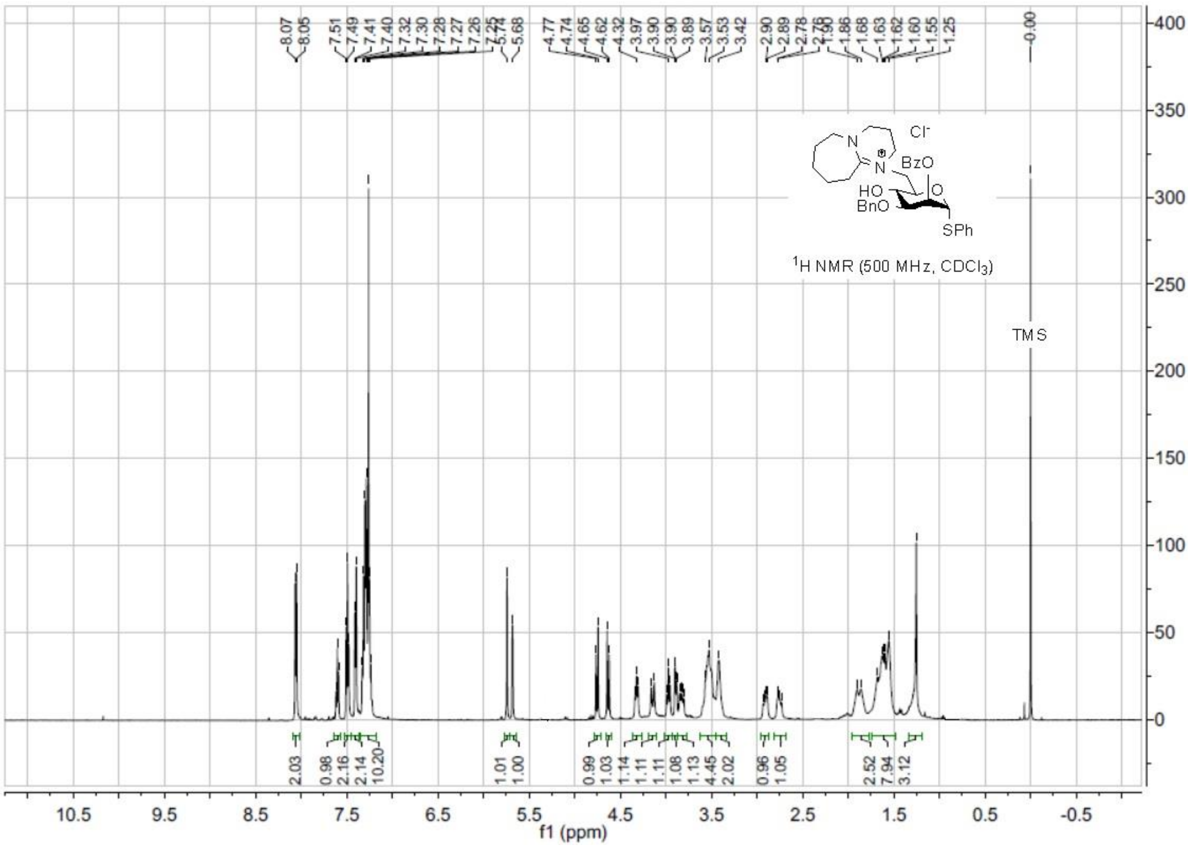

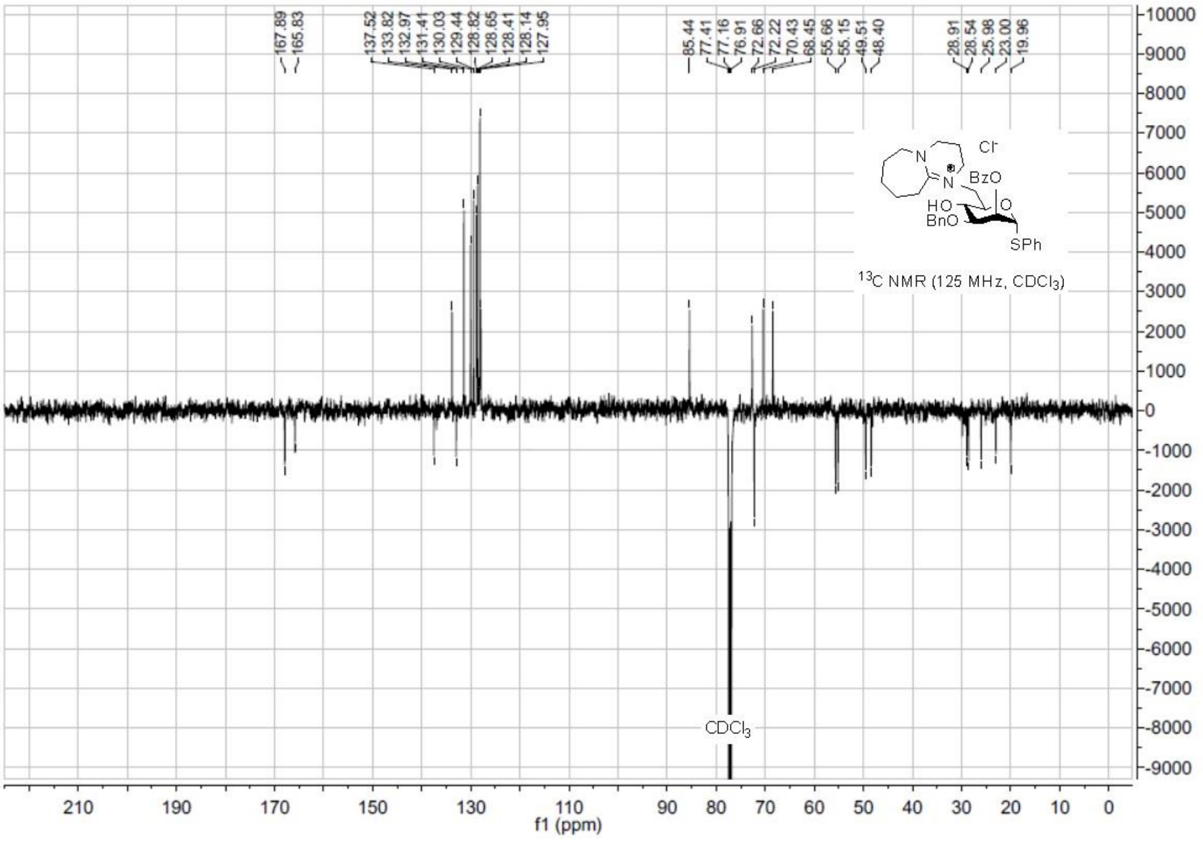

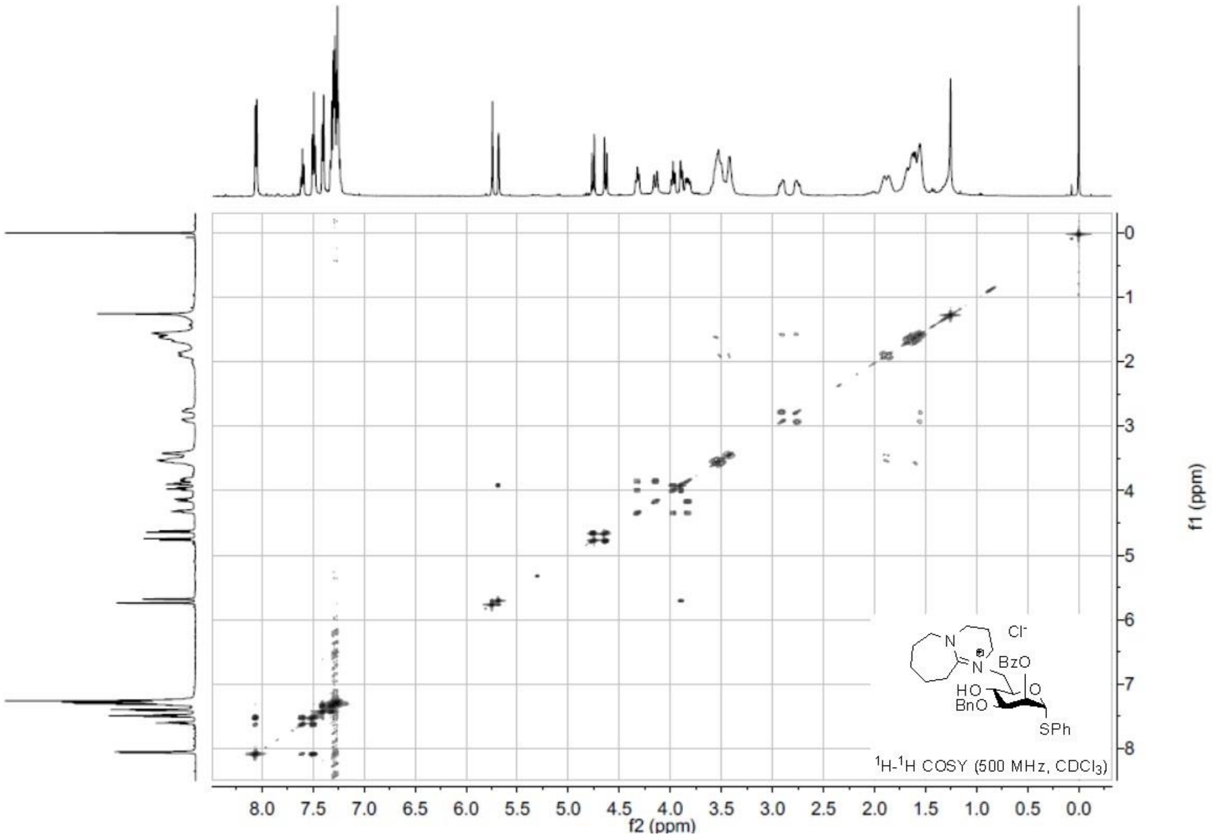
Cl⁻
N
N
BzO
HO
O
BnO
SPh
1H-1H COSY (500 MHz, CDCl3)
f2 (ppm)
f1 (ppm)

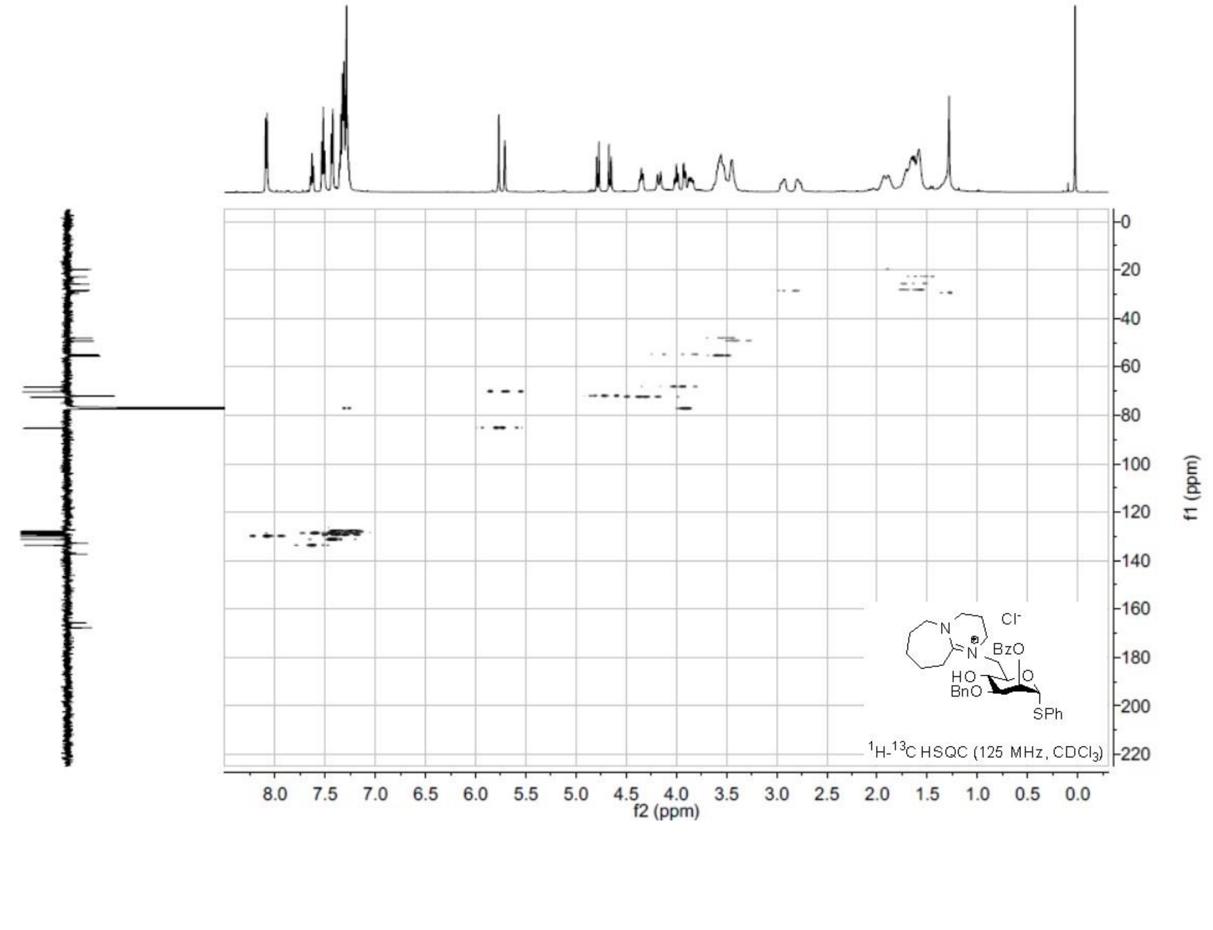
Cl⁻
N
N
BzO
HO
O
BnO
SPh
1H-13C HSQC (125 MHz, CDCl3)
f2 (ppm)
f1 (ppm)

^{1}H and ^{13}C NMR spectra of compound **53**

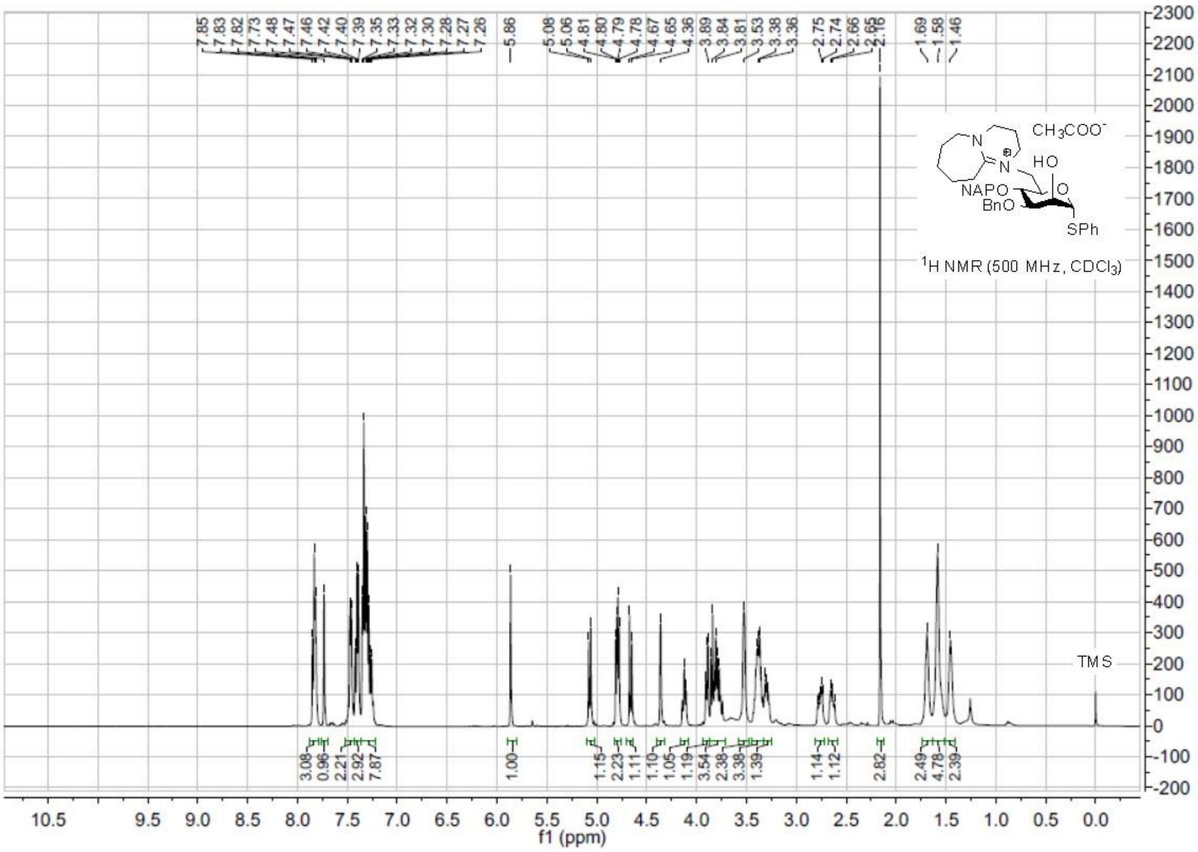

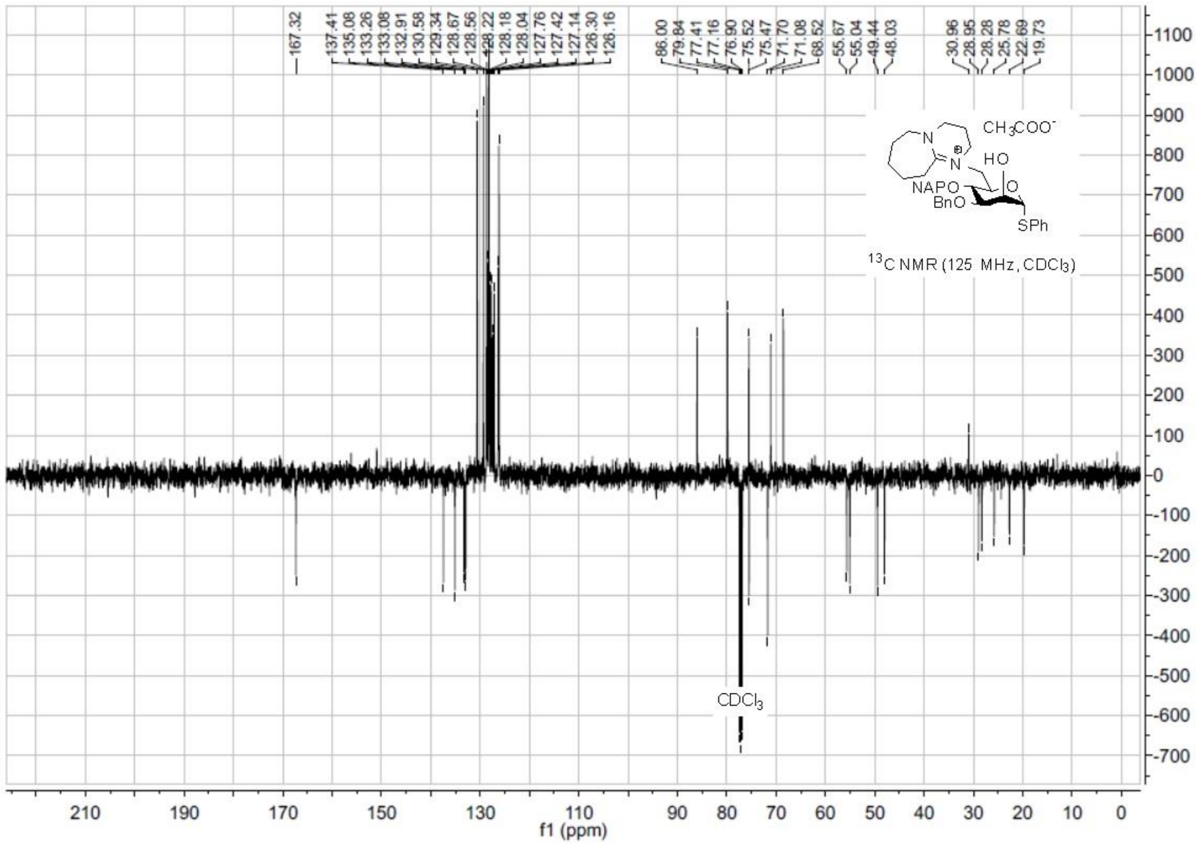

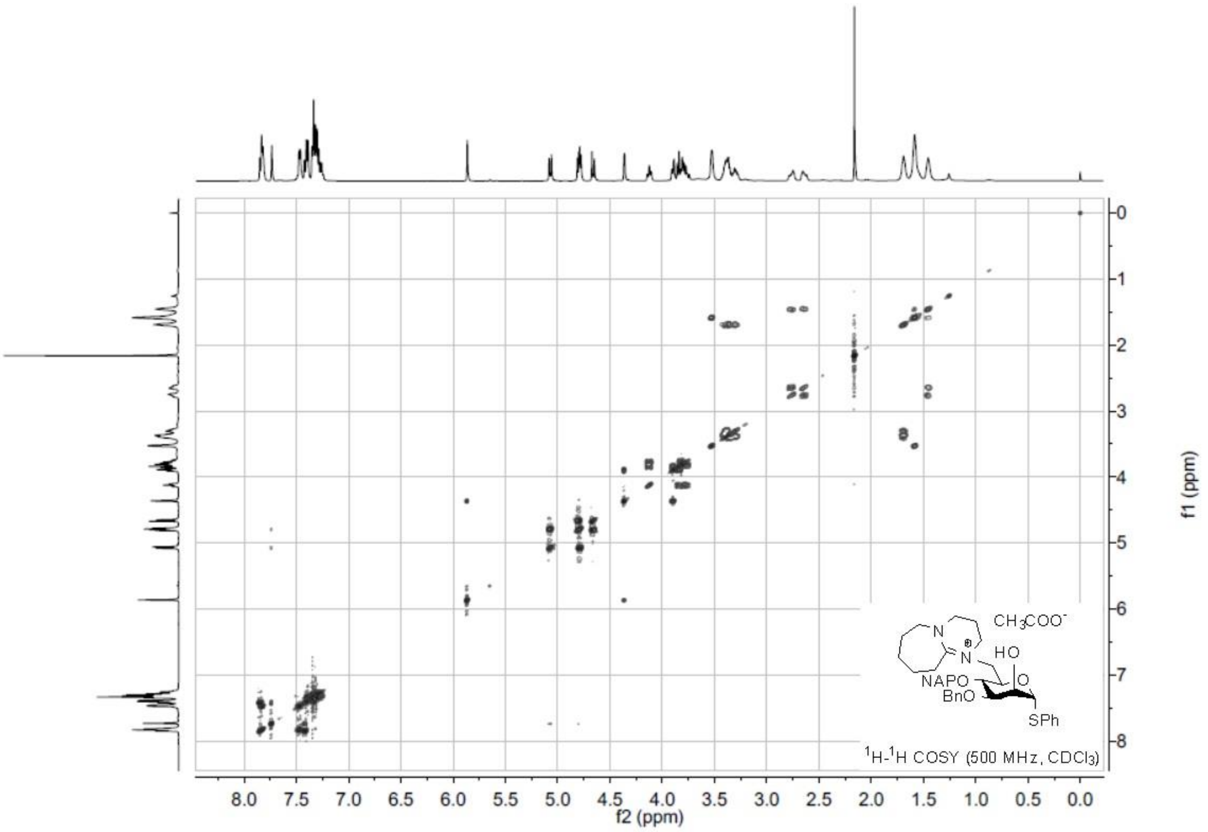
CH3COO-
HO
NAPO
BnO
SPh
1H-1H COSY (500 MHz, CDCl3)
f2 (ppm)
f1 (ppm)

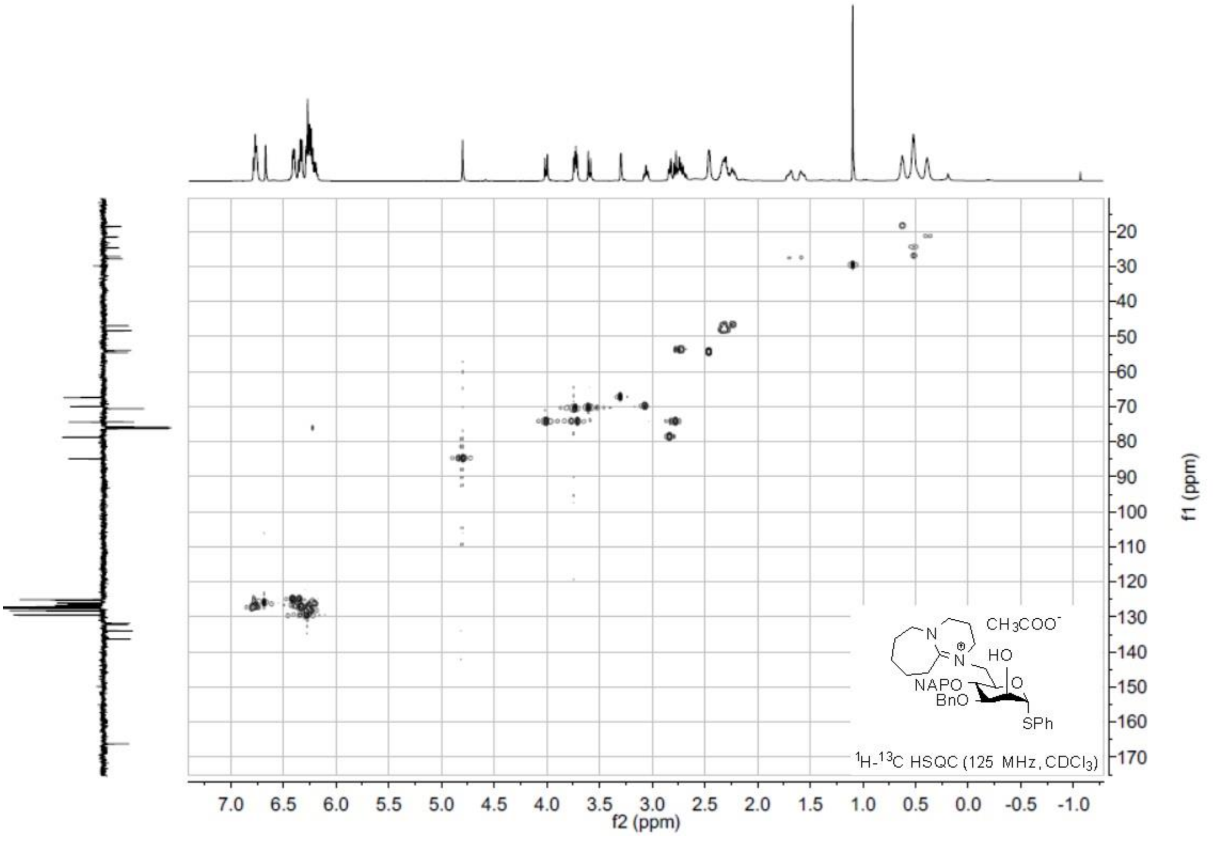
CH3COO-
HO
NAPO
BnO
SPh
1H-13C HSQC (125 MHz, CDCl3)
f2 (ppm)
f1 (ppm)
